# The Long-Term Efficacy of “Social Buffering” in Artificial Social Agents: Contextual Affective Perception Matters

**DOI:** 10.3389/frobt.2022.699573

**Published:** 2022-09-15

**Authors:** Imran Khan, Lola Cañamero

**Affiliations:** ^1^ Embodied Emotion, Cognition, and (Inter-)Action Lab, School of Physics, Engineering, and Computer Science, University of Hertfordshire, College Lane, Hatfield, United Kingdom; ^2^ ETIS Lab, CY Cergy Paris University—ENSEA—CNRS UMR8051, Cergy, France

**Keywords:** artificial agents, affective perception, affective interaction, social allostasis, social bonds, homeostasis, social buffering, stress

## Abstract

In dynamic (social) environments, an affective state of “stress” can be adaptive and promote agent wellbeing, but maladaptive if not appropriately regulated. The presence of (and interactions with) affect-based social support has been hypothesised to provide mechanisms to regulate stress (the “social buffering” hypothesis), though the precise, underlying mechanisms are still unclear. However, the hormone oxytocin has been implicated in mediating these effects in at least two ways: by improving social appraisals and reducing the short-term release of stress hormones (i.e., cortisol), and adapting an agent’s long-term stress tolerance. These effects likely facilitate an agent’s long-term adaptive ability by grounding their physiological and behavioural adaptation in the (affective) social environment, though these effects also appear to be context-dependent. In this paper, we investigate whether two of the hypothesised hormonal mechanisms that underpin the “social buffering” phenomenon affect the long-term wellbeing of (artificial) social agents who share affective social bonds, across numerous social and physical environmental contexts. Building on previous findings, we hypothesise that “social buffering” effects can improve the long-term wellbeing of agents who share affective social bonds in dynamic environments, through regular prosocial interactions with social bond partners. We model some of the effects associated with oxytocin and cortisol that underpin these hypothesised mechanisms in our biologically-inspired, socially-adaptive agent model, and conduct our investigation in a small society of artificial agents whose goal is to survive in challenging environments. Our results find that, while stress can be adaptive and regulated through affective social support, long-term behavioural and physiological adaptation is determined by the contextual perception of affective social bonds, which is influenced by early-stage interactions between affective social bond partners as well as the degree of the physical and social challenges. We also show how these low-level effects associated with oxytocin and cortisol can be used as “biomarkers” of social support and environmental stress. For socially-situated artificial agents, we suggest that these “social buffering” mechanisms can adapt the (adaptive) stress mechanisms, but that the long-term efficacy of this adaptation is related to the temporal dynamics of social interactions and the contextual perception of the affective social and physical environments.

## 1 Introduction

### 1.1 Background

The ability for a (social) agent to make contextually-relevant decisions depends on her perception of that context. Among other things, this is dependent on an agent’s affective state, the contextual cues in her (physical and social) environment, and the (types of) interactions available to her. This affective contextual perception plays an important role in the adaptation of behaviours, and is essential (particularly in dynamic environments) in facilitating the long-term wellbeing and autonomy of any adaptive (biological and artificial) agent. For these autonomous agents, adaptation to dynamic external environments is made more complex when the environment itself is (sometimes exclusively) comprised of other autonomous social agents that are also continually adapting their interactions. In these cases, the (real-time) affective perception, adaptation of behaviours and decision-making must also account for the affective interactions and dynamics of the social environment as part of their adaptive process: a seemingly-natural process in biological social agents (Schulkin, 2011).

One consistent theme in these biological (both human and non-human) systems is that individuals who exist as part of a society, form affective social relationships, and who positively interact with other social agents, report improved adaptability, wellbeing, and survival in dynamic (stress-inducing) environments (Schülke et al., 2010; Cameron et al., 2009; Holt-Lunstad et al., 2010). One prominent hypothesis to explain this phenomenon is known as “social buffering” (Kikusui et al., 2006; Cohen and Wills, 1985). This hypothesis posits that social support can provide a mechanism to mediate the maladaptive (psychological, physiological, and behavioural) “stress” responses (Hostinar, 2015) that occur during, and in anticipation of, threatening or stressful situations, and this socially-grounded regulation of “stress” facilitates the long-term wellbeing (that is, maintaining the stability of an organism’s internal milieu (Bernard, 1878) through homeostatic processes (Cannon, 1929)) of social agents with affective social support.

Given that stress can play a significant adaptive role in regulating homeostasis via physiological and behavioural adaptation (McEwen et al., 2015; McEwen and Wingfield, 2003), appropriate regulation of its adaptive (as opposed to its maladaptive (Hannibal and Bishop, 2014)) effects can propose significant advantages for socially-supported agents. Though the underlying mechanisms of “social buffering” are likely multi-faceted (Hostinar, 2015) and contextually-dependent (Sapolsky et al., 2000; Abbott et al., 2003)—making them difficult to elucidate in natural systems (Uno et al., 2002)—the hormone oxytocin has been hypothesised to mediate these effects in (at least) two ways: by increasing the valence of affective bond partners (Kirschbaum et al., 1993), and by “buffering” the activation of an internal stress (autonomic nervous) system (Ditzen and Heinrichs, 2014; Uno et al., 2002).

**FIGURE 1:**
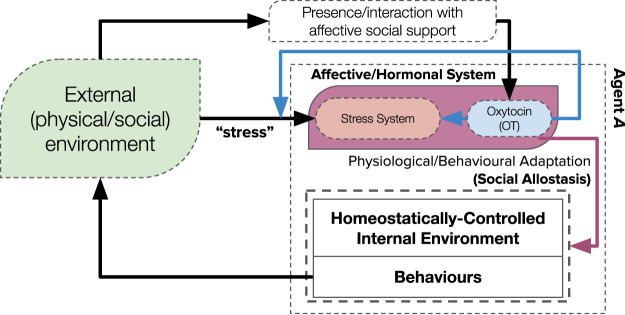
A theoretical framework for how the hypothesised mechanisms of “social buffering” that we investigate in this paper (blue and red lines) underpins a mechanism of “social allostasis,” *via* an intermediary hormonal system. Perceived stressful environments result in activation of an internal stress system (McEwen et al., 2015): positive interactions with affective social support releases oxytocin, which has numerous stress-regulatory effects (“social buffering” (Kikusui et al., 2006)). This affective hormonal system then adapts the internal homeostatic mechanism *via* physiological and behavioural adaptation.

The adaptive effects (on physiology and behaviour) presented by both “stress” and the “social buffering” phenomenon likely underpin an adaptive process known as “(social) allostasis” (McEwen and Wingfield, 2003; Sterling, 2020; Schulkin, 2011): a re-imagining of classical theories of homeostasis. Social allostasis describes an anticipatory or predictive adaptation of stability-seeking homeostatic mechanisms: by integrating prior and current (internal, external and social) information to adapt physiology and behaviours in anticipation of changing environments. Where homeostasis describes the maintenance of vital physiological parameters to an ideal (or a range of) set points—correcting deficits through error-correcting negative feedback loops—allostasis proposes that the homeostatic mechanisms (including the range of set points that ensures stability) can be (and, indeed, are) adjusted as a predictive or anticipatory process. These anticipatory adjustments of homeostatic mechanisms—likely mediated by hormones (such as cortisol and oxytocin) (Sterling, 2020)—aim to minimise (potential) future internal errors before they need to be corrected. It can therefore be considered as a “second-order” mechanism that adapts the adaptive (homeostatic) mechanism: though ambiguity of its precise definition still remains (Ramsay and Woods, 2014). Nevertheless, the “social buffering” phenomenon and its associated (hormonal) mechanisms can be appropriately integrated into the framework of social allostasis (Figure 1); proposing a biologically-plausible framework of social adaptation for (artificial) social agents (Sterling, 2020) which, in turn, can significantly contribute to our understanding of social adaptation of biological agents.

The remainder of the paper is set out as follows: we continue this section by describing some related work in Section 1.2 and present our research questions and hypotheses in Section 1.3. Section 2 presents a complete description of our simulation environment and agent model used in our investigation. Section 3 describes our experimental set up and presents our results. We discuss our findings further in Section 4, summarise our conclusions in Section 5, and highlight some of the work’s limitations and future directions in Section 6.

### 1.2 Related Work

Addressing social adaptation for embodied (socially-)adaptive agent models remains an ongoing area of research, with a range of approaches being considered. This includes affect-based behavioural adaptation in human-agent (both physical and virtual) interactions (Hiolle et al., 2014; Tanevska et al., 2019; Ghafurian et al., 2019; Youssef et al., 2015); imitation of others (Breazeal et al., 2005; Doering et al., 2019); and social learning through both physical (robot) (Bartoli et al., 2020) and virtual (Le et al., 2020, 2018) partners, as well as in multi-agent systems (Tampuu et al., 2017; Yonenoh et al., 2019; Pérez et al., 2017). Recent work has also considered models of higher-order cognitive functions: adapting behaviours by inferring the intention or affective state of others (Pieters et al., 2017; Görür et al., 2017), or by perspective taking of other social agents (Trafton et al., 2006).

Work in human-robot interactions (HRI) have also investigated the adaptive role of (the formation of) affective social bonds between humans and artificial (physical) agents, including work with both children (Belpaeme et al., 2012; Cañamero and Lewis, 2016; Ros et al., 2011; Ligthart et al., 2019) and adults (Vollmer et al., 2018; Shiomi et al., 2017; Andreasson et al., 2018; Hiolle et al., 2012; Willemse and Van Erp, 2019) across numerous types of embodiments (see the survey by (Leite et al., 2013)). In all of these works, however, the effects and analysis of these social bonds have been at a *dyadic* level (a human-agent pair bond), as opposed to the group or *social* level. Attempting to address those limitations, our previous work has investigated some of the adaptive effects of affective social bonds in a society of artificial (virtual) agents (Khan et al., 2020, 2019; Khan and Cañamero, 2021).

While work in HRI has also investigated the effects of social interactions on “stress,” this work has largely focused on how these interactions affect the “wellbeing” of the *human* actor (see (Ling and Björling, 2020) for a review), but not the artificial agent. For instance, (Aminuddin et al., 2016; Willemse et al., 2017; Shiomi and Hagita, 2021; Felnhofer et al., 2019), assessed numerous psychological and/or physiological measures related to “stress” during and after interactions with a robotic partner, with (Willemse and Van Erp, 2019) assessing the effects after partners had established an (affective) bond with their robot partner. Despite some recent work that assessed how an embodied model of “stress” affected the (compulsive) behaviours and wellbeing of a robot, (Lewis and Cañamero, 2019), more work is still required to understand how the adaptive properties associated with “stress” (and its underlying mechanisms) can affect (positively or negatively) the “wellbeing” of (virtual or physical) artificial agents.

### 1.3 Motivation, Research Questions, and Hypotheses

As noted by (Paiva et al., 2014), one common theme in these different approaches towards social adaptation is that their mechanisms of learning, prediction, and action are, in some way, inspired by biological systems. Despite its biological plausibility, models for socially-adaptive embodied agents have yet to consider the underlying mechanisms of “social buffering” (and “social allostasis” more generally) as part of their (bio-inspired) approaches. Given the challenges in studying the hypothesised hormonal mechanisms of “social buffering” in biological systems (Uno et al., 2002), artificial models can be used to abstract and study some of these effects *in silico* to further understand their function in biological systems. As initial steps to address this limitation, we have previously conducted investigations into the adaptive effects of both “stress” and one element associated with the “social buffering” phenomenon (*via* oxytocin’s effects on social salience modulation) using a society of artificial agents (Khan et al., 2020).

To address some of the limitations in the existing (biological and artificial agent) literature, the aim of the study presented in this paper is to investigate the effects of (two elements of) the “social buffering” phenomenon on viability (wellbeing) management and social behavioural dynamics. Specifically, we focus on two hypothesised hormonal effects that mediate this phenomenon: oxytocin’s effects on improving the valence of social partners (Hennessy et al., 2009) (i.e., the “social salience” modulation element), and oxytocin’s effects on “buffering” the internal stress system (Heinrichs et al., 2003)) (i.e., the stress tolerance modulation). Building on the findings of these “social buffering” effects in biological (Kikusui et al., 2006; Cohen and Wills, 1985) and artificial (Khan et al., 2020) societies, the two hypotheses (H1 and H2) for this study are as follows:

(H1): Social buffering would provide significant advantages (in the long term) to the wellbeing of a group of agents that share affective social bonds, when compared to groups without social bonds.

Our research questions stemming from this hypothesis are:

• RQ1.1: What is the effect that the social salience (“bond valence”—BV) modulation element of social buffering has on internal viability management?
• RQ1.2: What is the effect that adding the stress tolerance (threshold) modulation element of social buffering to the social salience modulation element has on (internal) viability management (particularly in the long term)?

**(**H2**)**: Social buffering would have significant effects on the type of social behavioural dynamics of groups.

Our research questions stemming from this hypothesis are:

• RQ2.1: What is the effect of the social salience modulation element of social buffering on the social behavioural dynamics of the group, in terms of positive (grooming) and negative (aggression) social behaviours?
• RQ2.2 What is the effect that adding the stress tolerance (threshold) modulation element of social buffering to the social salience modulation element has on the social behavioural dynamics of the group, in terms of positive (grooming) and negative (aggression) social behaviours?

Based on observations in humans which report mixed efficacy associated with the “buffering” of internal stress systems (Doom et al., 2017; Hostinar, 2015), we predict that we will see larger wellbeing improvements associated with oxytocin’s modulation of social salience (model type BV), than on its role modulating stress tolerance (model type BV+ST).

We conduct our investigation using a small, rank-based society of artificial agents whose goal is to “survive” (i.e., maximising their viability) through the maintenance of their internal (“physiological”) environment via homeostatic mechanisms. To test our hypotheses, we model numerous hypothesised effects associated with cortisol and oxytocin that underpin the “social buffering” phenomenon; and test their incremental effects on agent wellbeing and behaviours across three different social contexts (related to the agents that have social support available) and three dynamic physical environments (related to resource availability).

## 2 Agent Simulation

### 2.1 Simulation Environment

**FIGURE 2:**
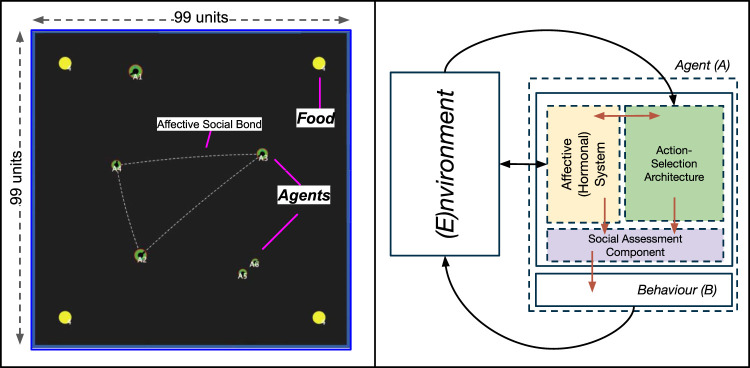
Left: A screenshot of our simulated environment, consisting of two types of resources: Food (yellow spheres) and Agents (green discs). The dotted line between agents indicates agents that share an affective social bond. The size of agents visually represents their social status (rank). Right: A top-level view of the components of the embodied agent model, with arrows indicating interactions between different components. These are described further in Section 2.2.1).

The simulation environment was developed using the NetLogo platform, version 5.3.1 (Wilensky, 1999), modelled as a simple, enclosed, two-dimensional world (of size 99 × 99 units). Mirroring our previous environments (Khan et al., 2020, 2019), the environment consisted of two types of objects (resources): autonomous *Agents*, and *Food*. A screenshot of the complete environment can be seen in Figure 2. The model is publicly available and can be found in our Supplementary Material S1.

**Agents** (represented as discs, Figure 2) are artificial entities whose actions are driven by the regulation of an internal, homeostatically-controlled “physiology” consisting of two competing needs: *Energy* and *SocialNeed*. This physiological regulation is achieved through an embodied agent model called the Action-Selection Architecture, discussed in Section 2.2.1) which all agents are endowed with.

Agents perceive the world through a fixed field-of-vision (20 units in radius by 80°), and interact with the world by either randomly *wandering* (an appetitive behaviour) through the environment at a rate of *speed* (defined as the number of units moved at each time step, and calculated using Eq. 10a) through the environment in the absence of external stimuli, or performing one of two consummatory behaviours: *Eating* food resources or *Touching* (either positively or negatively) other agents (Section 2.3). We describe these behaviours, and present visual examples, in Section 2.3.

**Food** (Figure 2, yellow spheres) constitutes a physical resource that can be acted on by agents (i.e., agents can *Eat* food). In our simulation, all food resources have the same maximum amount of “nutrition” (4 units), which decreases at a fixed rate (0.01 units per time step) when an agent is acting on it (i.e., *Eating* it). When an agent is not acting on (*Eating*) a food resource, its nutritional amount increases by 0.001 per time step (up to its maximum nutritional value): mirroring basic food “replenishment” in natural systems. The size of a food resource is dynamic, and equal to its nutritional availability (i.e., a food with 2 units of nutrition available is 2 units in size).

### 2.2 Agent Model

#### 2.2.1 Action-Selection Architecture

**FIGURE 3:**
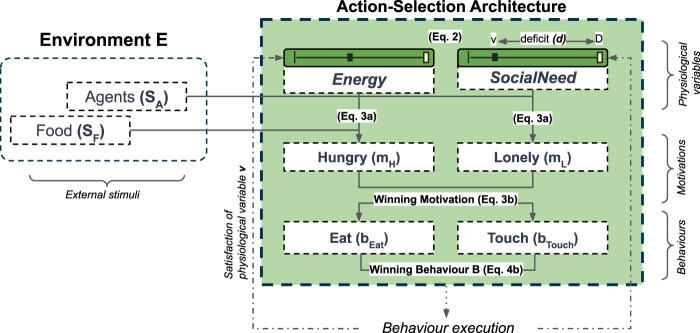
An overview of the Action-Selection Architecture which selects behaviours to regulate the two internal (physiological) variables *Energy* and *SocialNeed*. Numbers in parenthesis correspond to relevant equations in this section. Further details are provided in Table 1.

Following the approach originally proposed by (Cañamero, 1997) and used previously in our research group (Lones et al., 2017; Lewis and Cañamero, 2019; Cañamero and Lewis, 2016; Khan et al., 2020), the goal of our artificial agents is to maintain the “stability” (i.e., the viability (Ashby, 1954)) of their internal environment (the internal milieu (Bernard, 1878)), through the homeostatically-controlled regulation of two competing internal needs: *Energy* and *SocialNeed* (Figure 3).

Taking this approach, an agent’s actions (behaviours) are driven by an internal agent model (referred to here as the Action-Selection Architecture, or ASA) which consists of three connected layers: internal physiological needs **v**, motivational states **m**, and behaviours **b** (Table 1). The ASA selects from a set of behaviours to minimise the deficits (i.e., the errors) of these two internal needs to maximise an agent’s long-term well-being (i.e., maintaining viability). This approach to action-selection constitutes a classic, ethology-inspired (Tyrell, 1993) “Two-Resource Problem” (McFarland and Spier, 1997): a standard, minimally-complex approach to action-selection problems which has been used in previous studies of adaptive behaviours (Kiryazov et al., 2013; Lowe et al., 2010; Lewis and Cañamero, 2019; Montebelli et al., 2008).

**TABLE 1:** Relationship between internal “physiological” variables **v**, its relevant internal motivation **m**, the external stimuli **S** relevant to each internal variable, and behaviour **b** associated with each motivation OT: Oxytocin. CT: Cortisol. *: Only for agents with affective social bonds. **: Opposing effects based on behaviour: CT decreases (-) if *Groom*; CT increases (+) if *Aggression*. *TactInt* (Tactile Intensity) and *Speed*_*t*_ are discussed in the Agent Model section. All parameter values are apriori values taken from previous investigations (Khan et al., 2020; Khan and Cañamero, 2021).

Internal variable	Energy	SocialNeed
Loss Rate *γ*_*v*_	0.003 × 2 × (Speed_*t*_)	0.003
Motivation (m)	Hungry (*m*_*H*_)	Lonely (*m*_*L*_)
Stimuli (S)	Food (*S*_*F*_)	Agent (*S*_*A*_)
Behaviour (b)	Eat (*b*_Eat_)	Touch (*b*_Touch_) (Touch_Groom_, Touch_Aggression_)
Physiological Effect on Actor A	**v**: +0.003/time step	OT: +0.003 × TactInt* v: +0.05 × TactInt
Physiological Effect on Recipient R	N/A	OT: +0.003 × TactInt CT: +/- 0.003 × TactInt **

The first internal need (which we also call internal variables) is *Energy*, which is analogous to a physical need to consume physical resources (i.e., to maintain blood glucose levels). The second, called *SocialNeed*, is akin to a “psychological” (as well as physiological (Morrison, 2016)) need for tactile social contact. Both internal needs remain in the range 0–1, with an “ideal” set point of the upper limit (1). Each of these needs experience a small deficit at each time step (seen in Table 1), and agents are driven to maintain each variable as close to their respective ideal set points as possible. In this agent model, *Energy* is a survival-critical variable: if it drops to its minimum value (0), an agent is no longer viable and will “die.” We describe the complete mathematical details of our agent model below.

At the start of each time step **t**, each of the two internal variables **v**, *Energy* (*v*_1_) and *SocialNeed* (*v*_2_), experience a small loss at a rate of (*γ*_*v*_):

$$v_{n,t}=v_{n,t-1}-\gamma_{v}$$ (1)

The loss rate of *SocialNeed* is a preset, static value (*γ*_2_ = 0.003). Conversely, the loss rate of *Energy* is non-static: it is modulated by an agent’s movement speed (i.e., her “metabolic” activity; see Eq. 10a). We summarise this in Table 1.

Next, the deficits, or errors (**d**) of each of the internal variables **v** are calculated as the difference between each variable’s current value **v**_**t**_ and its “ideal” (or attractor) value **D**, respectively:

$$d_{v,t}=\text{D}-v_{n,t}$$ (2)

In line with the cue-deficit model proposed by (McFarland and Spier, 1997): each of these internal deficit values **d** are combined with the perception of external stimuli **S** relevant to correcting each of the deficits, to calculate the “intensity” of two internal motivations (**m**): *Hungry,* (*m*_*H*_) and *Lonely* (*m*_*L*_). In our agent model, the corresponding stimuli are *Food* (***S***_***F***_, to correct *Energy* deficits), and *Agents* (*S*_*A*_, to correct *SocialNeed* deficits) (see Table 1). Therefore:

$$m_{t}=d_{t}+\left(d_{t}\times S_{i}\right)$$ (3a)

$$M_{t}=\max\left(m_{\text{H}},m_{\text{L}}\right)$$ (3b)

Each motivational intensity remains in the range 0–1, with the motivation returning the highest value selected as the most “urgent” (winning) motivation **M**. Finally, the winning behaviour **B** is calculated by combining the intensity of the winning motivation **M** with the physiological effects *ω* that each of the behaviours **b** has on satisfying the internal variable **v** associated with that particular motivation. In the present model, each behaviour only has physiological effects on a single internal variable respectively, resulting in a 1:1 mapping between motivations and behaviour (Table 1). Concretely, to **Eat** (*b*_Eat_) when an agent is **Hungry** (*m*_*H*_), or **Touch** (*b*_Touch_, either *Groom* or *Aggression* which is described in Section 2.2.2) when the winning motivation is **Lonely** (*m*_*L*_) (Table 1).

$${\text{b}}_{t}=M_{t}\times \omega_{bv}$$ (4a)

$${\text{B}}_{t}=\max\left(b_{\text{Eeat}},b_{\text{Touch}}\right)$$ (4b)

Agents satisfy their current motivation by performing the winning behaviour either by moving to an available resource or wandering through the environment until a resource is available. When either of the error-correcting behaviours (Eat or Touch) have been performed, the value of the relevant internal variable is updated at a context-dependent rate (Table 1). In this two-resource problem of action-selection, agents are always motivated to perform one of these two actions at every time step *t*.

#### 2.2.2 Social Assessment Component

The Social Assessment Component (Khan et al., 2019), or SAC, provides an affective appraisal of the social environment prior to the execution of the winning behaviour **B**, which contextually adapts the (type of) behaviour that an agent performs. The SAC calculates the relative “value” (called AgentVal, *χ*) of agents it perceives in the (local) environment, to determine:

1) **which** agent to socially interact with when the winning behaviour is *Touch.*
2) whether to perform a socio-positive (**Groom**) or socio-negative (**Aggression**) behaviour when the winning behaviour is *Touch.*
3) whether to *approach* or *avoid* a (preoccupied) food resource when the winning behaviour is *Eat.*

This is calculated by combining three points of information: 1) an acting agent’s (*A*) affective (hormonal) state, 2) the (local) social status of other agents *R* in the environment (i.e. agents that it can immediately perceive), and 3) the existence and quality of an affective social bond between agents. Using this information, the SAC calculates a “trade-off” between affective and (local) social information—an abstraction of the flexible social decision-making seen in biological systems (Mielke et al. (2020); Asakawa-Haas et al., (2016); Gerber et al.,(2020); Mielke et al., (2020, 2018); Asakawa-Haas et al., (2016); Gerber et al., (2020))—prior to the execution of (social) behaviours. We define the components of the SAC below.

**Social Status (Rank)**: In our model, an agent’s social status (or **social rank**) K corresponds to her status in a hierarchical society. It is a predetermined, fixed value; modelled as a normalised value between 0 and 1 (with 0 being the lowest-ranked and increasing in *1/n* increments, where **n** is the total number of different ranks in a society). An agent’s social status (rank) loosely corresponds to the degree of “control” she has in the environment. By default, higher-ranked agents have priority access to resources, and are favoured by others for positive social interaction, which is calculated using Eq. 6.

**Affective Social Bond**: An affective social bond is defined as a pre-existing, mutually-positive affective social relationship between two agents (for instance, a parent–offspring relationship). In our model, it is represented as a fixed Boolean flag (**Ξ**_**AB**_ = **1** if a bond exists, else **0**).

**Bond Strength (Dyadic Strength Index)**: Affective social bonds also have a “strength” associated with them, which we refer to as the *Dyadic Strength Index* (DSI, **ϒ**). This is a bi-directional value that stays in the range 0–2, used to model and measure the affective quality of a social bond between two agents. Higher DSI values denote a mutually-strong affective bond between agents *A* and *B*, while a low value denotes a mutually-weak affective bond between them. The strength of these bonds are subject to a small decay rate (*μ*_ϒ_ = 0.9997) at each time step when a social interaction has not taken place between bonded agents (Table 1). In other words, in absence of any social interaction that reinforces or strengthens a bond, social bonds between agents become “weaker”.

The SAC then works as follows: Firstly, agent *A* calculates the relative rank difference (normalised between -1 and +1 in 0.25 increments) and each agent *B* she perceives:

$$\Delta K_{\left(AB\right)}=K_{A}-K_{B}$$ (5)

Negative Δ*K* values correspond to agent *B* outranking agent *A*, and vice-versa for positive values. The relative rank difference is then combined with the presence and quality of an affective bond between agents *A* and *B*.

$$\chi_{B}=\underset{\begin{array}{c} \text{Relative Rank}\\ \text{Difference} \end{array}}{\underset{︸}{\Delta K_{\left(AB\right)}}}+\underset{\begin{array}{c} \text{Affective Bond Status} \end{array}}{\underset{︸}{\left[\Xi_{AB}\times \left({ϒ}_{\Xi ,AB}\times {\text{OT}}_{A}\right)\right]}}$$ (6)

When no bond exists between agents *A* and *B*, ***χ***_**B**_ = Δ*K*_(*AB*)_. As we see in Eq. 6, the bond strength ϒ is further modulated by **OT**_*A*_. Here, OT stands for “oxytocin”; one of two simulated hormones with numerous modulatory effects in the agent model. We describe this in further detail in the next section. Combining all of these parameters, *χ*_*B*_ can take the range -1 to +3.

#### 2.2.3 Affective (Hormonal) System and Effects

**FIGURE 4:**
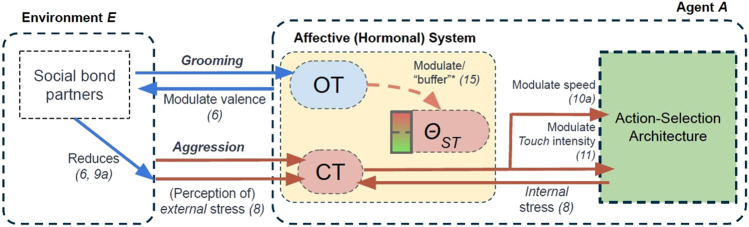
An overview of the hormonal mechanisms modelled in the Affective (Hormonal) System of our agent model. OT = Oxytocin. CT = Cortisol. *θ*_ST_ = Stress Tolerance. Number in parentheses is the equation(s) that correspond to our modelling of specific effects. * = Effect accounted for in our second experiment only. Descriptions of these effects are seen in this section, and summarised in Table 2.

Building on our own previous work (Khan et al., 2019, 2020; Khan and Cañamero, 2021), and also following the approach that has been used extensively in related approaches (Lewis and Cañamero, 2019; Lones et al., 2017; Cañamero and Lewis, 2016; Montebelli et al., 2008; Iizuka and Di Paolo, 2008), our agent model accounts for several biologically-inspired abstractions of hormonal mechanisms that act as (hormonal) modulators on an agent’s physiology and behaviours. These hormonal mechanisms are inspired by two biological hormones: **cortisol** (CT) and **oxytocin** (OT). However, we do not propose that our computational abstractions are a precise modelling of the complete biological hormones themselves. Rather, these models aim to capture the dynamics of some of the (hypothesised) mechanisms of these hormones in biological systems, and, in line with the approach of (Cañamero, 2019), aim to capture some features of “affective cognition” grounded in the hormonal modulation of an underlying action-selection model. Nevertheless, these abstractions are grounded in biological studies (see Table 2 and subsequent subsections), which we propose can support the generalisability of our model to biological systems. These hormonal effects have been illustrated in Figure 4 and summarised in Table 2.

**TABLE 2:** Overview of the two hormones in the Affective (Hormonal) System in our agent model: describing how each hormone is secreted (increased), inhibited (reduced), and the modulatory effects they have on agent physiology or behaviour. Number in parentheses are corresponding equation(s) for each effect. Cells in blue denote our abstractions of the two “social buffering” effects that we investigate.

Hormone	Increased *via*	Reduced *via*	Modulatory Effects on Agent model	Abstracted biological effect
Cortisol (CT)“Stress hormone”	Receiving Aggression (Section 2.2.3.1)	Performing *Aggression* (Section 2.2.3.1)	Modulates agent speed/*Energy* expenditure (Eq. 10a)	Cortisol increases energy metabolism Brillon et al. (1995)
	Internal and External Stress (Eq. 8)			
			Modulates intensity of *Touch* interactions (*Groom*, *Aggression*) (Eq. 11)	“Stress” increases intensity of tactile interactions.
Oxytocin (OT)“Social hormone”	Receiving/Performing *Grooming* (Eq. 14)	No (tactile) interaction with social bond partner (Eq. 14)	Modulates valence of social bond partners Eq. 6	Oxytocin improves bond partner valence Crockford et al. (2014)
			Modulates internal “stress tolerance” (Eq. 15)	Oxytocin “buffers” HPA-axis activation DeVries et al. (2003)

The first “hormone” in our model, **cortisol** (CT), represents a stress-related hormone: released as a function of (perceived internal and external) stressors (including receiving negative social interactions from agents). The second hormone, **oxytocin** (OT) can be considered a “social” hormone which is released as a function of positive (tactile) social interaction. As we describe below (and also summarised in Table 2), our modelling of oxytocin is used to capture two of the hypothesised “stress-regulating” effects of social support. A third component of our affective system, called **Stress Tolerance**, simply describes the amount of cortisol an agent can withstand in her physiology, where transgression of this tolerance results in her undergoing an affective state of “stress.” In this model, it can be considered a simple abstraction of an autonomic nervous system (Kyrou and Tsigos, 2009; McEwen et al., 2015).

##### 2.2.3.1 Cortisol

In our model, cortisol (CT) is a stress-related (Bollini et al., 2004) hormone that plays an adaptive role (Schulkin, 2003) on agent physiology and behaviour (Table 2). CT levels are dynamic, and either “secreted” (increased) or “inhibited” (reduced) in an agent at a rate of ***γ***_**CT**_: calculated as a function of an agent’s perceived *internal* and *external* stress. *Internal* stress relates to an agent’s physiological deficits and *external* stress relates to the perceived availability of external resources (i.e. her perceived level of “control” of her physiology and environment (Bollini et al., 2004)). For agent A, at time step *t*, changes in CT levels are calculated as follows:

$${\text{CT}}_{t}={\text{CT}}_{t-1}+\gamma_{\text{CT}}$$ (7)

$$\text{where }\gamma_{\text{CT}}=\left(\underset{\text{Internal Stress}}{\underset{︸}{\bar{d_{v}}}}-\underset{\text{External Stress}}{\underset{︸}{\frac{{\hat{S}}_{\text{agents}}+{\hat{S}}_{\text{food}}}{2}}}\right)\times w$$ (8)

Here, $\bar{d_{v}}$ is the mean deficit (Eq. 2) of the two physiological variables (*Energy* and *SocialNeed*), indicating how far an agent is from her ideal physiological state. External stress $\left({\hat{S}}_{\text{agents}}+{\hat{S}}_{\text{food}}\right)/2$ is calculated as the mean “availability” of external stimuli (*Agents* or *Food*) that an agent perceives. In other words, it is a value which represents the perceived level of “certainty” or “control” of the external environment (where a lack of certainty or control results in higher “stress” (Sapolsky, 2004)). Using the affective appraisal of other agents (*χ*, calculated in Eq. 6), resource “availability” is calculated as follows:

$${\hat{S}}_{\text{agents}}=S_{\text{agents}}\times \left(1-\chi_{B}\right),\text{ and }$$ (9a)

$${\hat{S}}_{\text{food}}=1\text{ when }\chi_{B}\geq 0\text{ else }0$$ (9b)

A positive resulting value of *γ*_CT_ results in CT being increased in an agent, and a negative value results in CT being inhibited in an agent. Finally, **w** = 0.005, a scalar parameter used to regulate the sensitivity of *γ*_CT_, and which was tuned to our specific environmental conditions.

CT has two modulatory effects on our agent model. These have been summarised in Table 2 and illustrated in Figure 4. Firstly, inspired by its effects on energy metabolism (Brillon et al., 1995; Careau et al., 2008), CT modulates the default movement speed (Speed_0_ = 0.5 units/time step) of an agent. This increased movement speed proportionally depletes an agent’s *Energy* at a rate of *γ*_*E*_:

$${\text{Speed}}_{t}={\text{Speed}}_{\text{0}}\times \left(1+\text{CT}\right)$$ (10a)

$$\gamma_{E}=\gamma_{E0}\times \left(2\times {\text{Speed}}_{t}\right)$$ (10b)

Secondly, CT directly modulates the “intensity” at which tactile interactions (*Touch*: either *Touch*_Groom_ or *Touch*_Aggression_) are performed by an agent. Specically: the more CT present in an agent’s physiology, the “stronger” the tactile interaction (TactInt):

$$\text{TactInt}=b_{\text{Touch}}\times \text{CT}$$ (11)

where $b_{b_{\text{Touch}}}$ is the intensity of the winning *Touch* behaviour (calculated by Eq. 4b).

Abstracting dynamics of affective touch (Morrison, 2016), the intensity of this tactile interaction affects both the acting agent *A* and recipient *R* of the tactile interaction. For agent *A*, it satisfies her physiological *SocialNeed* variable (*v*_2_), and reduces her own level of CT, proportional to *TactInt*:

$$v_{2}={v_{2}}_{t-1}+\left(\text{TactInt}\times c\right)$$ (12)

$${\text{CT}}_{t}={\text{CT}}_{t}-\text{TactInt}$$ (13)

For the recipient agent *R*, CT is either inhibited or secreted—dependent on the type of tactile interaction received—proportional to the *TactInt* received by agent *A*:

$${\text{CT}}_{\text{R}}=\left\{\begin{array}{cc} {\text{CT}}_{\text{R}}-{\text{TactInt}}_{A}\times o,\; & \text{if }{\text{Touch}}_{\text{Groom}}\\ {\text{CT}}_{\text{R}}+{\text{TactInt}}_{A}\times o,\; & \text{if }{\text{Touch}}_{\text{Aggression}} \end{array}\right.$$

Finally, the strength of any existing social bond (the *DSI*, ϒ, described in Section 2.2.2) between agents *A* and *R* is also proportionally strengthened or weakened, dependent on the type of tactile interaction received:

$${ϒ}_{\text{AR}}=\left\{\begin{array}{cc} {ϒ}_{\text{AR}}+\text{TactInt}\times i,\; & \text{if }{\text{Touch}}_{\text{Groom}}\\ {ϒ}_{\text{AR}}-\text{TactInt}\times i,\; & \text{if }{\text{Touch}}_{\text{Aggression}} \end{array}\right.$$

Finally, all scalar values in the equations above (*c* = 0.1; *o* = 0.3, *i* = 0.5) are apriori values used to regulate the values of respective equations, which were tuned to our specific environment prior to previous investigations (Khan et al., 2020, 2019; Khan and Cañamero, 2021).

##### 2.2.3.2 Stress Tolerance

Each agent is endowed with a Stress Tolerance value (*θ*_ST_, between 0 and 1): an abstraction of the “autonomic nervous system” in biological systems (Kyrou and Tsigos, 2009; McEwen et al., 2015)). This Stress Tolerance value simply determines how much of the stress hormone CT an agent can withstand in her physiology before she becomes “stressed”. All agents start with the same *Stress Tolerance* (*θ*_ST(d)_ = 0.5).

Combined with the affective assessment of social agents (Eq. 6), this state then determines whether the winning behaviour *Touch* will be performed in a positive (Groom) or negative (Aggression) manner.

When CT levels do not transgress an agent’s Stress Tolerance (CT $<\;\theta_{\text{ST}}$ ), all *Touch* behaviours with other agents are socio-positive (Touch_Groom_). However, when CT ≥ *θ*_ST_ (i.e. an agent is “stressed”), agents adapt their *Touch* behaviour—performing either Touch_Aggression_ or Touch_Groom_ based on their affective appraisal of the other agent (Eq. 6). Therefore, when agent *A* is driven to *Touch* another agent *B*:

$$b_{\text{Touch}}=\left\{\begin{array}{cc} {\text{Touch}}_{\text{Groom}}\; & \text{if CT}<\;\theta_{\text{ST}}\\ {\text{Touch}}_{\text{Groom}}\; & \text{if CT}\geq \;\theta_{\text{ST}}\text{and}\chi_{B}\geq 1\\ {\text{Touch}}_{\text{Aggression}}\; & \text{if CT}\geq \;\theta_{\text{ST}}\text{and}\chi_{B}<1 \end{array}\right.$$

##### 2.2.3.3 Oxytocin

The second hormone in our model, oxytocin (OT), is a socially-influenced hormone (Quintana and Guastella, 2020) that models numerous dynamics of the biological hormone in natural systems. In our present model, it is secreted as a function of (positive) social interactions (Uvnas-Moberg and Petersson, 2005; Carter, 2014) and has two modulatory effects which serve as abstractions of two hypothesised mechanisms that underpin the “social buffering” phenomenon (Kikusui et al., 2006; Cohen and Wills, 1985). First, increased OT increases the valence of social support: reducing the perceived stress associated with the external environment ((Taylor, 2006; Hennessy et al., 2009)). Second, it modulates (or “buffers”) an agent’s internal stress tolerance (akin to activation of the sympathetic nervous system, (Heinrichs et al., 2003); described in Section 2.2.3.2).

Abstracting effects of “affective touch” (Ellingsen et al., 2016; Morrison, 2016), OT is “secreted” in both the acting (*A*) and recipient (*R*) agents after a positive tactile interaction. The rate of secretion is proportional to the intensity of the tactile interaction (TactInt, Eq. 11) performed by *A*. In other words, the “stronger” the positive tactile interaction, the more OT released in both agents. At all other times, OT experiences a small decay (*μ* = 0.005) per time step. Therefore, for both agents *A* and *R*:

$$OT_{t}=\left(OT_{t-1}-\mu_{\text{OT}}\right)+{\text{TactInt}}_{A}$$ (14)

As mentioned above, our modelling of OT has two modulatory effects related to its hypothesised, stress-reducing effects. Firstly, OT modulates the valence of affective bond partners it perceives: a mathematical abstraction of OT’s hypothesised role on preferential attention to (and improved valence of (Taylor, 2006; Hennessy et al., 2009)), affective bond partners in natural systems (Shamay-Tsoory and Abu-Akel, 2016). This modulatory effect is described in Eq. 6: which then has an effect on 1) the perception of external (environmental) stress (Eq. 9a, Figure 4), and 2) the affective appraisals of social agents when performing *Touch* behaviours (Section 2.2.2).

OT’s second effect is that it “buffers” an agent’s internal tolerance to (physiological) stress (the **Stress Tolerance** discussed in Section 2.2.3.2, by modulating an agent’s default Stress Tolerance ${\theta_{\text{ST}}}_{0}$ proportional to her OT level:

$$\Theta_{{\text{ST}}_{t}}=\Theta_{{\text{ST}}_{0}}\times \left(0.5+{\text{OT}}_{t}\right)$$ (15)

where the default Stress Tolerance ${\theta_{\text{ST}}}_{0}=0.5$ . Since OT stays in the range 0–1, the possible range for Θ_ST_ = 0.25–0.75.

### 2.3 Agent Perception and Behaviours

Agents have a fixed 80° field-of-vision of length 20 units. Agents perceive resources when they fall within this range. In absence of a relevant resource, agents randomly **wander** through the environment, initially at a default rate of 0.5 units per time step, which is modulated by the CT in its physiology (E). When the presence of a stimuli motivates an agent to perform a behaviour (Eq. 3a), they move towards that resource to perform one of their two consummatory behaviours:

• **Eat** is performed on food resources by stopping to take “bites” of food, satisfying their internal *Energy* variable at a fixed rate (+0.01 per time step) until the motivation has been satisfied.
• **Touch** is a tactile interaction between two agents and encapsulates both the socio-positive *Groom* and socio-negative *Aggression* behaviours, which is determined by the affective state of the acting agent, and their affective relationship with the other agent (Section 2.2.3.2). In both instances, Touch behaviours are executed in a single time step and have contextual effects on the satisfaction of the internal *SocialNeed* of the actor, hormone secretion/inhibition and affective bond quality (Table 1).

We provide short visual examples of these behaviours in videos found in our Supplementary Material S1.

## 3 Experiment and Results

### 3.1 Experimental Conditions

Experiments were conducted in the simulation environment described in Section 2. Following the approach in our previous work (Khan et al., 2020, 2019; Khan and Cañamero, 2021), we used a society of six artificial agents, where three agents share affective social bonds and the remaining three are unbonded. We have found that this group size (six agents) to be a suitable number of agents that 1) allows the society to be split into multiple (non-dyad) groups, 2) permits for the appropriate level of complexity for analysis, and 3) allows for analysis at the levels of the overall society, the sub-groups, and the individual agents (which we call the macro, meso, and micro-levels, respectively). A summary of our experimental conditions can be found in Table 3, with detailed descriptions below.

**TABLE 3:** Table summarising the three experimental parameters and a brief description of each experimental condition. Coloured cells correspond to the illustration of the model types in Figure 6. BV = Bond Valence; BV+ST = Bond Valence + Stress Tolerance. World conditions are illustrated in Figure 5. Equations refer to our modelling of each respective effect. Agent number refers to her hierarchical rank (1 = highest, 6 = lowest).

Model types	
Control	Agent model does not include any of OT’s effects.
Type BV	OT modulates the valence of social support (Eq. 6); reducing the perception of external stress (Eq. 8)
Type BV+ST	Type BV effects and OT modulates internal stress tolerance (Eq. 15)
**Bond Combinations**	
A	Agents A1-A2-A6 bonded; Agents A3-A4-A5 unbonded.
B	Agents A3-A4-A5 bonded; Agents A1-A2-A6 unbonded.
C	Agents A4-A5-A6 bonded; Agents A1-A2-A3 unbonded.
**World Conditions**	
**Name**	**Description**
Static (STA)	Static environment where four food resources are fixed in the corners of the environment (Figure 5, left).
Seasonal (SEA)	Dynamic environment where food resources steadily changes every 1,000 time steps in one-food increments (Figure 5, centre).
Extreme (EXT**)**	Dynamic environment where food resources suddenly changes every 1,000 time steps increments (Figure 5, right).

**Model Types (3)**: Our investigations were conducted using two separate models, describing the type of OT mechanism(s) that our agents were endowed with, plus a control model. In our first experiment, OT *only* modulated the valence of social bond partners (Eq. 6), reducing the perceived stress from the environment (Eq. 8). We refer to this as the Type BV (Bond Valence) model. In our second experiment, OT retains these effects and also modulates an agent’s internal *Stress Tolerance* value (Eq. 15; Figure 4. We refer to this as the **Type BV+ST** (Bond Valence + Stress Tolerance) model. Our **Control** condition described an agent model that did not include any of OT’s effects, but did retain all of the CT mechanisms we described in Section 2.2.3.1.

**Affective Bond Combinations (3)**: For each of these model types, we tested three different combinations of agents who shared affective social bonds in the society, with the other three agents without them. These combinations referred to agents of different social ranks (from A1, the highest-ranked, to A6, the lowest-ranked), and are inspired by relationships in biological systems. The three combinations were as follows: Bond Combination A consisted of agents A1-A2-A6 sharing affective social bonds, constituting an (allo-)parental relationship; with Bond Combination B (A3-A4-A5 bonded) and Bond Combination C (A4-A5-A6 bonded) constituting middle-ranked and lowest-ranked “close-kin” relationships respectively.

**FIGURE 5:**
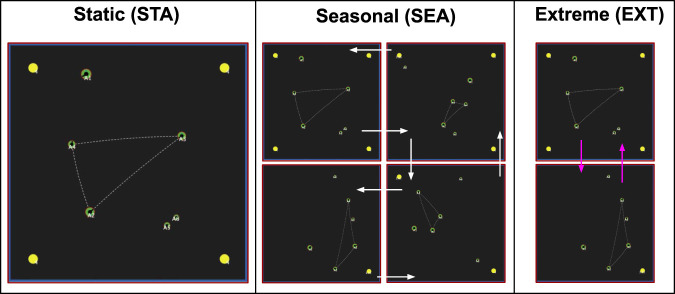
Screenshots of the three environments that our experiments were conducted in. Yellow spheres = food resources. Green discs = agents. Left: The Static (STA) environment, with four fixed food resources in each corner of the simulated environment. Centre: The *Seasonal* (STA) environment. After 2000 time steps, food resources (yellow spheres) incrementally decrease by one resource every 1,000 time steps (inner white arrows); and then increase at the same rate (outer white arrows). Right: The *Extreme* (EXT) environment. After 2000 time steps, food resources immediately change from four to one, back to four, every 1,000 time steps. The size of the disc represents an agent’s rank (the higher the rank, the larger the agent).

**World Conditions (3)**: We conducted our experiments in three different physical environments (Figure 5. These physical environments (which we also call world conditions) refer to the dynamics of food availability, and represent different degrees of physically-challenging (and thus increasingly-stressful) environments. Following from previous work (Khan et al., 2019), the base world condition (the *Static* (STA) environment) consists of four food resources, fixed in the four corners of the environment. The second world condition, *Seasonal* (SEA) was a dynamic environment where, after 2000 time steps, the number of food resources steadily changed every 1,000 time steps (which we refer to as a “season”): changing from four→one→four in one-food increments. This loosely corresponds to biological seasons, where food steadily becomes more and less abundant in fixed time intervals. Our third environment, *Extreme* (EXT), was a dynamic environment where changes in food resources were more severe: changing from four to one resource instantaneously across the same time intervals. This represented more “extreme” changes in the physical environment (i.e. such as an unpredictable change in the ecosystem which results in severe food shortages). The specific details of these environmental changes can be found in Table 4.

**TABLE 4:** Availability of food across different “seasons” in each of the world environments illustrated in Figure 5. Food availability is colour-coded for visualisation purposes: green indicates periods where maximum number of food resources 4) are available, and red indicates periods where minimum number of food resources 1) are available.

	
Time step	Start	0	2001	3001	4001	5001	6001	7001	8001	9001	0,001	11,001	12,001	13,001	14,001
	End	2000	3000	4000	5000	6000	7000	8000	9000	10,000	11,000	12,000	13,000	14,000	15,000
World Condition	Season	1A/1B	2	3	4	5	6	7	8	9	10	11	12	13	14
Static															
Seasonal		4	3	2	1	2	3	4	3	2	1	2	3	4	3
Extreme		4	1	4	1	4	1	4	1	4	1	4	1	4	1

**FIGURE 6:**
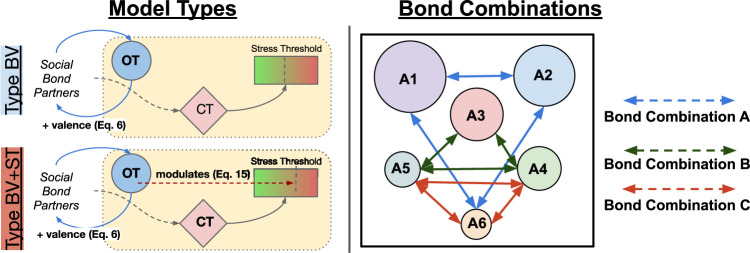
Left: The two different model types investigated in our experiments. Right: The three different combinations of agents that shared affective social bonds (Bond Combinations). Arrows between agents indicate agents that shared an affective social bond in each condition.

Experiments were conducted using a PC running Windows 10 64-bit operating system with an Intel Xeon CPU at 2.5 Ghz (8 threads) and 12 GB of RAM. Each experimental condition was simulated 20 times, with each run limited to 15,000 time steps (where one time step is defined as a single simulation update cycle). We have previously found this (Khan et al., 2020) to be a sufficient number of observations for statistical analysis in similar experimental set ups. The total run time for all experiments using this set up was approximately 70 h Figure 6.

### 3.2 Metrics

Agent performance was primarily assessed in terms of its “viability” (Ashby, 1954). Using the metrics proposed by (Avila-Garcia and Cañamero, 2004), we considered agent viability across numerous measures; by both accounting for the length of time (the quantity) of her viability, as well as how well an agent regulates her internal variables (the “quality” of stability).

The first metric, *Life Length* (LL), describes the number of time steps that agent *A* survives (i.e. keeps her *Energy* variable above 0) as a percentage of the total simulation run time (between 0% and 100%). It is calculated as follows:

$${\text{LL}}_{A}=\frac{t_{{\text{life}}_{A}}}{t_{\text{max}}}$$ (16)

where $t_{{life}_{A}}$ is the total number of time steps agent *A* kept her *Energy* above 0, and *t*_*max*_ is the maximum simulation run time (in this case, 15,000). The second measure, *Comfort* (CO) is one of two measures of “quality,” and measures the mean value (between 0 and 1) of an agent’s two internal variables (*Energy* and *Social*):

$${\text{CO}}_{A}:=\overset{t_{{\text{life}}_{A}}}{\underset{i=1}{\sum }}\frac{\left(1-\bar{d_{i}}\right)}{t_{{\text{life}}_{A}}}$$ (17)

where $\bar{d_{i}}$ is the mean of the two internal variables *Energy* and *SocialNeed*. The second measure of “quality”, *Physiological Balance* (PB) reports the homogeneity (between 0 and 1) of the satisfaction of both internal variables during an agent’s life.

$${\text{PB}}_{A}=\overset{t_{{\text{life}}_{A}}}{\underset{i=1}{\sum }}\frac{\left(1-\left(\left|d_{1}-d_{2}\right|\right)\right)}{t_{{\text{life}}_{A}}}$$ (18)

where (1 − (|*d*_1_ − *d*_2_|) denotes the absolute difference between the internal variables *Energy* (*d*_1_) and *SocialNeed* (*d*_2_).

In addition to these viability-related metrics, we reported data related to the agents’ internal hormone (OT and CT) and Stress Tolerance (Θ_*ST*_) levels, and the (temporal) distribution of social behaviours (*Groom* and *Aggression*. We also report rates of “intra-bond” (*Grooming* or *Aggression*) interactions: the number of either *Grooming* or *Aggression* that a bonded agent (or group of agents) performed towards other members of their bonded group, as a percentage of the total number of that particular type of interaction performed by that agent (group). Specifically:

$${\text{intraBond}}_{\text{Groom}}=\frac{{\text{Groom}}_{\text{A→B}}}{{\text{Groom}}_{\text{A→B}}+{\text{Groom}}_{\text{A→UB}}}$$ (19)

$${\text{intraBond}}_{\text{Aggression}}=\frac{{\text{Aggression}}_{\text{A→B}}}{{\text{Aggression}}_{\text{A→B}}+{\text{Aggression}}_{\text{A→UB}}}$$ (20)

where Groom_*A*→*B*_ is the number of grooming interactions performed by agent *A* on another bond partner *B*, and Groom_*A*→UB_ is the number of *Grooming* interactions performed on unbonded (UB) agents. This definition is identical for *Aggression* interactions.

Data was captured at each time step at the individual agent level, and aggregated across all simulation runs. We use Pearson’s Correlation Coefficient to describe correlations between experimental variables. For between-group comparisons, we use one-way analysis of variance (ANOVA). Statistical significance is reported at the 0.05 level. We supplement our quantitative results with qualitative reporting of agent and group behaviour where appropriate.

### 3.3 Results

We first report results regarding our viability-related metrics (*Life Length* (LL), *Comfort* (CO), and *Physiological Balance* (PB)) across our different experimental conditions. We report results both at the aggregated society level, and on the performance of bonded agent groups, with some reporting of unbonded groups where appropriate.

#### 3.3.1 Viability Indicator Metrics

**FIGURE 7:**
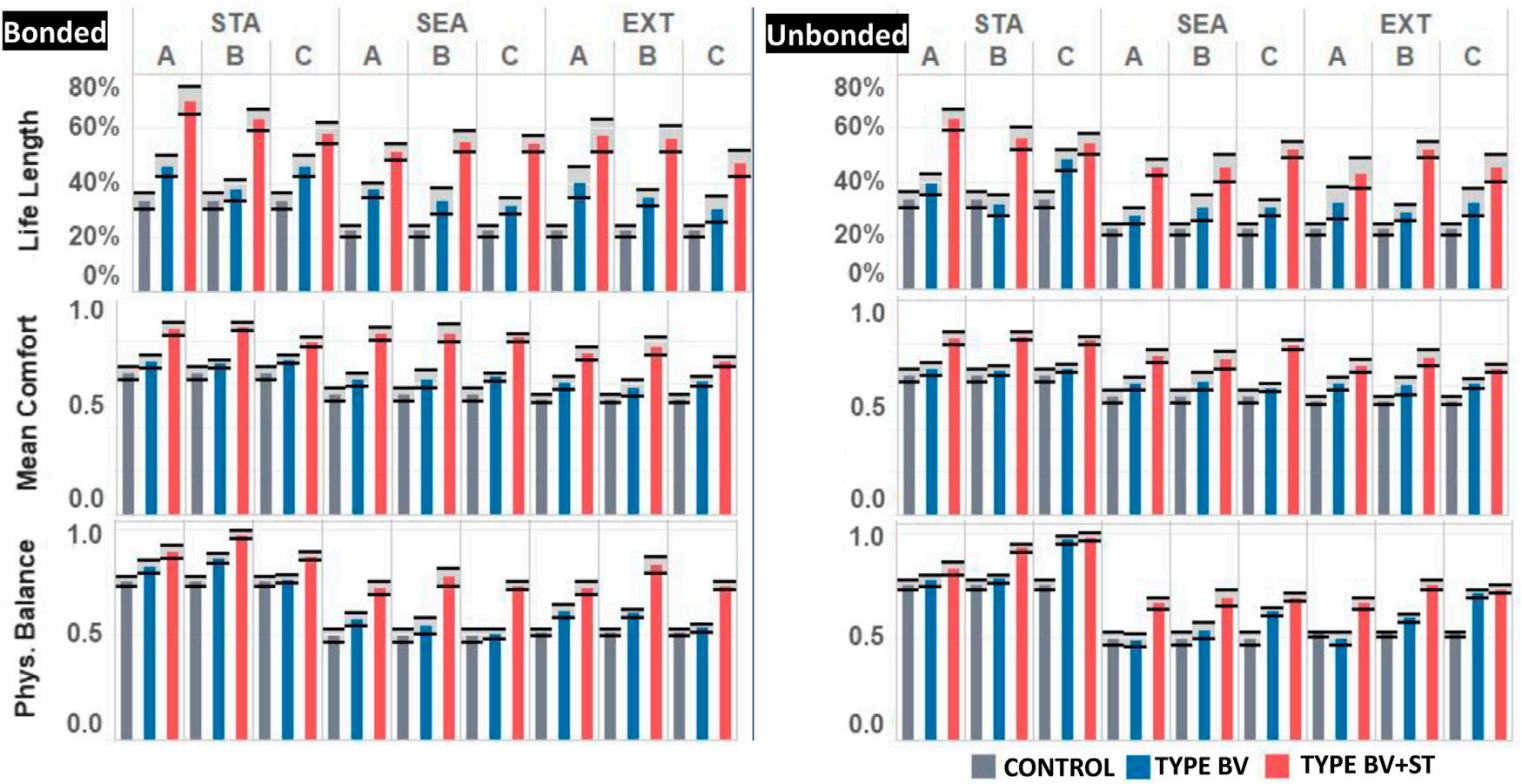
Results of the three viability-related metrics by model type (Control, Type BV, Type BV+ST): aggregated by bonded (Bo) and unbonded (Unb) agents across all world (STA: Static, SEA = Seasonal, EXT = Extreme) and Bond (A,B,C) conditions. Black bars show standard error of mean values.

Overall, compared to control, bonded agents across both the Type BV and Type BV+ST conditions reported significant improvements across all three viability indicator metrics (Life Length, Mean Comfort, and Physiological Balance). In general, Type BV+ST agents reported greater overall improvements in viability compared to Type BV agents. These results were reported at an aggregated level for bonded agent groups, as well at the society level. Figure 7 shows the mean results of the viability indicator metrics for bonded agents across all experimental groups.

**Life Length**: At an aggregated level, both Type BV and BV+ST models outperformed control groups across all experimental conditions, with Type BV+ST groups reporting statistically-significant differences across all conditions. Comparing between-groups, Type BV+ST groups survived for longer than Type BV across all environmental conditions: with LL improvements between 20% and 70% in STA world conditions, 56%–75% in SEA world conditions, and 37%–72% in EXT world conditions (*p* < 0.05 across all results). This improvement was seen for both bonded (+27%–+74%) and unbonded (+13%–+86%) agents in Type BV+ST conditions vs. Type BV conditions.

**Comfort**: We found statistically-significant improvements in CO for bonded agents in Type BV+ST conditions (STA: 0.82–0.85 (*p* = 0.035), SEA: 0.81–0.84 (*p* = 0.031, EXT: 0.73–0.80 (*p* = 0.025)) vs. Type BV agents (STA: 0.69–0.71, SEA: 0.62–0.63, EXT: 0.58–0.61). There was a non-significant difference between bonded agents in Type BV conditions and Control groups (*p* = 0.22), but a statistically-significant difference between Type BV+ST and Control (*p* < 0.01 for all world conditions and bond combinations), and Type BV+ST vs. Type BV (*p* = 0.02 − 0.042) models. These improvements were found in bonded agents (Figure 7, left), and to a lesser extent in unbonded agents (Figure 7, right). Overall, only bonded agents in the Type BV+ST groups reported consistent improvements in Mean Comfort vs. Control groups.

**Physiological Balance**: Bonded agents in both Type BV (STA: 0.78–0.80, SEA: 0.78, EXT: 0.76) and Type BV+ST (STA: 0.87–0.97, SEA: 0.72–0.77, EXT: 0.72–0.83) conditions reported statistically-significant improvements in PB vs. Control. Compared to Control, bonded agents reported improvements to PB in both Type BV (+6–21%, *p* = 0.031 − 0.21), with statistically-significant improvements across all conditions in Type BV+ST (+16–49%, *p* = 0.01 − 0.02) groups. For both model types, unbonded agents also experienced improvements in PB (Type BV: −2–+39%, *p* = 0.036 − 0.49; Type BV+ST: +11–43%, *p* = 0.022 − 0.036).

In sum, we found improved viability-related performance for bonded agents (and for some unbonded agents) in conditions where half of the society shared affective social bonds and were endowed with (at least) one of the stress-regulating effects. Comparing between models, groups endowed with the Type BV+ST model (those with two stress-regulating effects of OT) outperformed Type BV groups (endowed with one mechanism) across all viability-related metrics. This was seen across all environmental and bond conditions.

#### 3.3.2 Hormone and Stress Tolerance Levels

##### 3.3.2.1 Oxytocin

**FIGURE 8:**
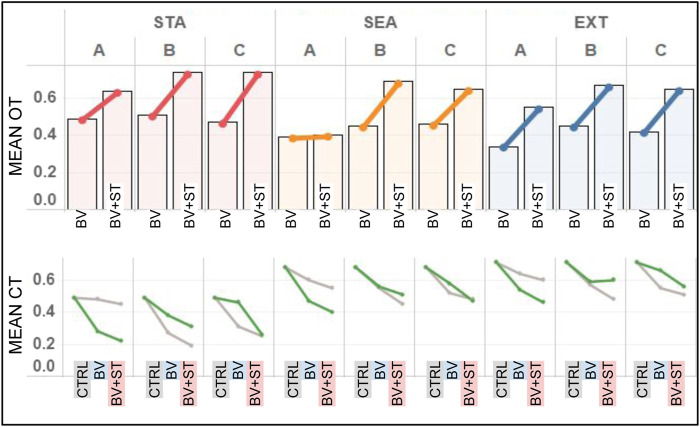
Results for the mean OT and CT levels across all experimental conditions. STA = Static, SEA = Seasonal, and EXT = Extreme world environments, respectively. Bond Conditions are denoted as A, B, and C, respectively. Top: Mean OT levels for bonded agents in both Type BV and Type BV+ST models. Bottom: Mean CT levels for bonded (green line) and unbonded (grey line) agents across Control (CTRL), Type BV, and Type BV+ST models.

In both Type BV and Type BV+ST groups, bonded agents reported the highest amount of oxytocin (OT) in *STA* world conditions (Figure 8). This result was found across all bond combinations. We found associations between the increasing difficulty of the physical environment (STA, SEA, EXT) and reduced levels of mean OT for bonded agents. This was seen across both Type BV (r = 0.763) and Type BV+ST (r = 0.693) groups.

Bonded agents endowed with the Type BV+ST model reported statistically-significant higher mean OT levels vs. Type BV societies across all environmental conditions (Bond A: +7%–75%, B: +46%–57%, C: +42%–57%, *p* < 0.01 for all conditions), with one exception (Bond A in *SEA* environments). These improvements in OT were reported despite no direct changes on OT “secretion” between these two models. Surprisingly, and despite their higher dominance ranks, agents in Bond A (Figure 8) reported the lowest mean OT levels (0.55–0.64) compared to lower-ranked Bond B (0.67–0.74) and C (0.65–0.74) respectively in the Type BV+ST groups.

##### 3.3.2.2 Cortisol

In Control groups, we found mean CT levels to be strongly inversely-correlated with an agent’s social rank across all environmental conditions (*STA*: r =0 .711, *SEA*: r = 0.826, *EXT*: r = 0.829). This correlation was not found in conditions using the Type BV or Type BV+ST models, when social bonds were accounted for.

In conditions using either the Type BV or Type BV+ST model, we found lower mean CT levels for both bonded and unbonded agents vs. Control (Figure 8, bottom). Increases in mean CT levels in these groups were associated with the increasingly-challenging world conditions (*STA*, *SEA* and *EXT*, respectively, Figure 8, bottom). Comparing the two groups, bonded agents with the Type BV+ST model reported statistically-significant reductions in mean CT vs. Type BV bonded agents across all bond combinations and environmental conditions (STA: -6 to -24% *p* = 0.013 − 0.025, SEA: -7 to -11%, *p* = 0.027 − 0.036, EXT: -5 to -6%, *p* = 0.033 − 0.047), with one exception (EXT, Bond B, +1%, not significant). In sum, Type BV+ST groups experienced significantly lower levels of mean CT when compared to both Control and Type BV groups.

**FIGURE 9:**
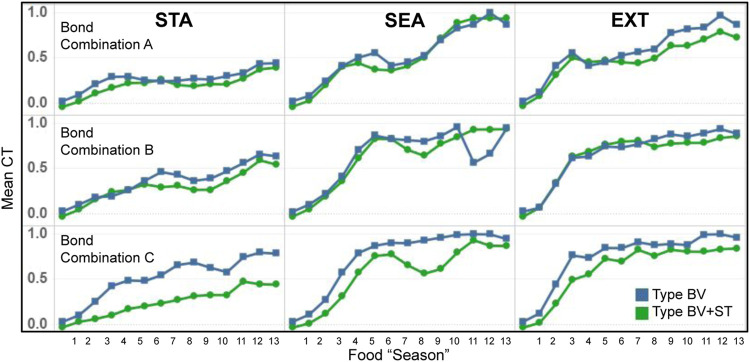
Temporal evolution of mean CT levels for bonded agents in Type BV (blue) and Type BV+ST (green) model conditions, across the different environmental conditions. STA = Static, SEA = Seasonal, EXT = Extreme world conditions, respectively.

As we had expected, we found that the evolution of mean CT levels to be associated with the dynamics of the three different physical environments (STA, SEA, EXT, Figure 9). While CT levels increased throughout the course of the experiment for both model types, the magnitude of these increases was significantly greater in the two dynamic conditions (SEA and EXT): roughly following the different dynamics of food availability (Table 4). Across all conditions, mean CT trends were similar between both Type BV and Type BV+ST groups, with the latter generally reporting lower mean CT levels in comparison.

Mean CT and Viability Indicators: At an individual agent level, we found a moderately-strong correlation between mean CT levels and mean survival time (LL) for agents with affective social bonds. This association was seen across both Type BV (r = 0.631) and Type BV+ST (r = 0.690) groups. In Type BV+ST groups, this correlation was weaker in *STA* environments for all Bond Conditions (A: r = 0.392, B: r = 0.453, C: r = 0.211), but stronger over *SEA* (A: r = 0.529, B: r = 0.704, c: r = 0.658) and *EXT* (A: r = 0.687, B: r = 0.723, c: r = 0.608) world conditions. We found no association between Mean Comfort (CO) and mean CT levels, but moderately-strong correlations between mean CT and Physiological Balance (PB) in the two dynamic environments (*SEA*: A: r = 0.841, B: r = 0.810, C: r = 0.748, *EXT*: A: r = 0.752, B: r = 0.776, C: r = 0.547).

#### 3.3.3 Stress Tolerance

**FIGURE 10:**
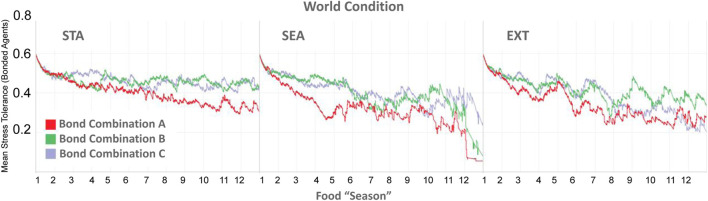
Temporal evolution of the internal Stress Tolerance (*θ*_*ST*_) values, aggregated for bonded agents in each bond condition. Bond A = Agents A1, A2, A6 bonded. Bond B A3, A4, A5. Bond C A4, A5, A6. Mean *θ*_*ST*_ for individual agents can be seen in Table 5.

As a reminder, in the Type BV+ST model, OT also modulated an agent’s internal Stress Tolerance (*θ*_*ST*_, Eq. 15), which had a default value of 0.5. Figure 10 shows the evolution of the mean *θ*_*ST*_ value across all three environmental conditions, aggregated for all agents in each bond condition. Mean *θ*_*ST*_ at the agent-level can be seen in Table 5. Overall, mean *θ*_*ST*_ for bonded agents corresponded to the environmental difficulty: *STA*: Bond A: 0.42, Bond B: 0.56, Bond C: 0.54; SEA: Bond A: 0.35, Bond B: 0.42, Bond C: 0.42, and EXT: Bond A: 0.33, Bond B: 0.51, Bond C: 0.42, respectively.

**TABLE 5:** Mean values of the *Stress Tolerance* for bonded agent in Type BV+ST models, aggregated across all simulation runs. Default *Stress Tolerance* for unbonded agents = 0.5. Red cells highlight agents that shared an affective bond in those conditions. STA, Static; SEA, Seasonal; EXT, Extreme world conditions, respectively.

World Condition	Bond Combination	A1	A2	A3	A4	A5	A6	Mean of Bonded Agents
STA	A	0.31	0.33	0.50	0.50	0.50	0.61	**0.42**
STA	B	0.50	0.50	0.60	0.54	0.53	0.50	**0.56**
STA	C	0.50	0.50	0.50	0.54	0.55	0.52	**0.54**
SEA	A	0.29	0.31	0.50	0.50	0.50	0.44	**0.35**
SEA	B	0.50	0.50	0.43	0.41	0.42	0.50	**0.42**
SEA	C	0.50	0.50	0.50	0.44	0.41	0.40	**0.42**
EXT	A	0.29	0.31	0.50	0.50	0.50	0.40	**0.33**
EXT	B	0.50	0.50	0.48	0.52	0.52	0.50	**0.51**
EXT	C	0.50	0.50	0.50	0.43	0.43	0.40	**0.42**

Bold values indicate mean Stress Tolerance value for agents who shared an affective bond in each condition.

Despite some agents (A1 and A2) in Bond Condition A being the highest-ranked in the society, agents in this bond condition reported the lowest mean *θ*_*ST*_ values across all conditions (A1, 0.29–0.31, and A2 0.31–0.33), with the lowest-ranked bond partner A6 adapting their *θ*_*ST*_ to significantly-higher values (0.40–0.61). Bonded agents in Bond Conditions B and C reported significantly less variance between their *θ*_*ST*_ values (0.2–0.7) than agents in Bond Condition A (0.11–0.30).

In summary, the adaptation of internal Stress Tolerance was not consistent between groups, but contextually-dependent on the specific bond combination and the degree of physical (environmental) challenge. Within bonded groups themselves, the adaptation of an agents’ internal Stress Tolerance was not consistent, with closer-ranked agents (Bond Conditions B and C) reporting less variance between bonded agents than bonded agents with larger rank differences (Bond Condition A).

#### 3.3.4 Social Interactions

##### 3.3.4.1 Intra-Bond Grooming and Aggression

We now report the rates of “intra-bond” socio-positive *Groom* and socio-negative *Aggression* interactions that occurred between agents who shared an affective social bond. As a reminder, the term “intra-bond” refers to interactions that take place between agents who share an affective social bond in each bond combination condition. The rate is defined by Eq. 19.

**Grooming**: Overall, we found intra-bond *Grooming* rates to approximately correspond to the mean social rank of bonded agents (Bond Conditions A, B, and C, respectively; Table 6) for both Type BV and Type BV+ST models. In other words, the higher the mean rank of the bonded group, the higher the amounts of intra-bond *Grooming*. Comparing Type BV and Type BV+ST agents, Type BV+ST agents reported significantly higher rates of intra-bond *Grooming* across all bond and world conditions (Table 6), with these differences most notable in Bond Conditions B and C.

**TABLE 6:** Rates of intra-bond *Grooming* and *Aggression*, determined as a percentage of overall *Grooming* and *Aggression* behaviours performed by bonded agents in each condition. STA, Static, SEA, Seasonal, EXT, Extreme world conditions.

		Model type			
		Type BV		Type BV+ST	
Bond Condition	World Condition	Grooming (%)	Aggression (%)	Grooming (%)	Aggression (%)
	STA	64	31	68	25
	SEA	60	42	63	27
A	EXT	60	45	65	23
	STA	37	78	54	69
	SEA	42	91	45	52
B	EXT	29	78	47	73
	STA	15	100	23	100
	SEA	17	100	28	100
C	EXT	22	100	35	100

However, we found inconsistent results between intra-bond *Grooming* rates and increased environmental challenges (STA, SEA, and EXT world conditions respectively). In Type BV groups, Bond Condition A reported their highest intra-bond *Grooming* rates in *STA* world environments (64%), Bond Condition B in *SEA* environments (42%), and Bond Condition C in *EXT* environments (22%). Similar results were reported by Type BV+ST agents (Bond Condition A: *STA* [68%], C: *EXT*: [35%]), with Bond Condition B reporting improved intra-bond *Grooming* in *STA* environments (54%).

Aggression: For all groups (including Control), overall *Aggression* rates at the society level were associated with the relative challenge of the world environment (Table 6). *STA* world conditions saw the lowest Aggression rates (Type BV: 7–9%, Type BV+ST: 6%–9%), with higher ranges seen in the *SEA* (Type BV: 12–13%, Type BV+ST: 10%–14%) and *EXT* (Type BV: 14–18%, Type BV+ST: 14%–18%) world conditions.

We found a moderately-strong correlation between the relative challenge of the world and the total number of *Aggression* interactions at a society level for all agents across Type BV (r = 0.526) and Type BV+ST (r = 0.661) groups. At the intra-bond level, however, across all bond groups and environmental conditions, bonded agents endowed with the Type BV+ST model reported lower intra-bond *Aggression* rates vs. Type BV. In other words, higher rates of *Aggression* interactions by Type BV+ST bonded agents were performed on agents with whom they did not share an affective social bond (unbonded agents), compared to the *Aggression* performed by those endowed with the Type BV model.

In sum, bonded agents with the Type BV+ST model reported higher rates of intra-bond *Grooming*, and lower rates of intra-bond *Aggression* compared to Type BV models. While no consistent association was found for socio-positive *Grooming* rates at an aggregated level, overall and intra-bond *Aggression* rates were associated with increasingly-challenging (i.e. more stress-inducing) world conditions.

##### 3.3.4.2 Temporal Distribution of Positive Social Interactions

**FIGURE 11:**
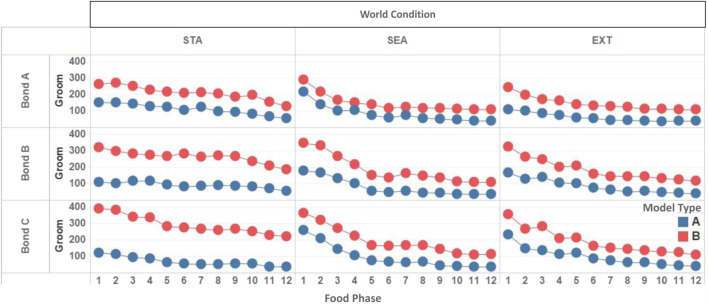
Temporal dynamics of total intra-bond *Grooming* between bonded agents in each world condition (STA = Static, SEA = Seasonal, EXT = Extreme) across 20 simulation runs, for both Type BV (blue) and Type BV+ST (red) models.

Compared to the Type BV group, bonded agents endowed with the Type BV+ST model saw an improvement in the absolute number of intra-bond *Grooming* interactions (Figure 11; Table 6). These improvements in the number of intra-bond *Grooming* interactions were most notable during the earlier stages in the world environments. In the Type BV group, 36% of all intra-bond *Grooming* occurred within the first two food “seasons” (2,000 time steps, when food remained unchanged) aggregated across all conditions. This was lower for bonded agents in the Type BV+ST group (with 24% of all intra-bond *Grooming* occurring during the same time period), despite reporting an increase in the overall *Grooming*.

In other words, higher rates of intra-bond *Grooming* were concentrated in earlier phases in Type BV models, with *Grooming* interactions in Type BV+ST models found to be more dispersed throughout the experimental run. While we expected *Grooming* to decrease in later phases as the environmental stressors increased in the dynamic physical environments (*SEA* and *EXT*), we found this to occur more in agents endowed with the Type BV model, but less so in agents with the Type BV+ST model.

**Early-Stage Grooming and Life Length**: We further analysed whether early-stage socio-positive interactions (defined as number of interactions within the first 2,000 time steps, where food availability was abundant in all conditions) between bonded agents were associated with improvements in long-term viability. While we found mixed, non-significant correlations between these variables in Type BV conditions, we found that in Type BV+ST conditions, the total amount of early-stage intra-bond *Grooming* was strongly correlated with mean Life Length across all bond conditions (Bond Condition A: r = 0.723, B: r = 0.772, C: r = 0.610), and similarly-strong correlations as world conditions increased in difficulty (Table 7).

**TABLE 7:** Results of r-values of Pearson’s Correlation Coefficient tests between the number of early-stage (i.e., before 2,000 time steps) intra-bond Grooming interactions and mean AgentVal∖Chi value across both model types, bond conditions and world conditions.

	Early-stage grooming × AgentVal						Early-stage grooming × Life length					
Model type		Type BV			Type BV+ST		Type BV			Type BV+ST		
WORLD/BOND CONDITION	A	B	C	A	B	C	A	B	C	A	B	C
STATIC	0.542	0.614	0.680	0.691	0.772	0.757	0.387	0.451	0.353	0.591	0.680	0.550
SEASONAL	0.551	0.601	0.578	0.811	0.745	0.785	0.427	0.224	0.623	0.710	0.759	0.633
EXTREME	0.696	0.753	0.732	0.838	0.850	0.848	0.586	0.375	0.332	0.802	0.798	0.605

**Early-Stage Grooming and Affective Perception**: We then performed a post-hoc analysis to assess the relationship between the number of early-stage (t < 2000 time steps) positive social interactions (*Grooming*) and the mean AgentVal (*χ*, the measure of affective perception) of bond partners. We present this relationship in Table 7 Overall, the higher the amount of early-stage *Grooming* between bond partners, the higher the mean affective perception (AgentVal, *χ*) of bond partners in the long-term. This relationship was significantly stronger in Type BV+ST models (where OT had a secondary effect on adapting the internal Stress Tolerance) than Type BV models where OT only increased the valence of social bond partners. We discuss the reasons for this in Sections 4.2, 4.3.

In sum, early-stage *Grooming* between bond partners was associated with improvements in mean Life Length for bonded agents with the Type BV+ST model across numerous physical and social environments. >From the post-hoc analysis, we also found a relationship between the increased difficulty of the physical environment and the affective perception of bonded agents in the Type BV+ST (but not Type BV) group. Unlike bonded agents with the Type BV model, positive social interactions between bonded agents in Type BV+ST models were significantly less affected by dynamic environmental conditions, with positive social interactions more evenly distributed across the experimental runs in the Type BV+ST groups.

## 4 Discussion

Our results found how social support, through some of the hypothesised hormonal mechanisms that underpin the “social buffering” phenomenon, resulted in significant improvements in viability for artificial agents who shared affective social bonds, across several stress-inducing environmental and social conditions. These artificial agents reported significant viability improvements vs. control when their agent models accounted for either one or two of oxytocin’s (OT’s) hypothesised stress-regulatory effects: modulating the valence of social support (Type BV models in our experiments), and “buffering” the internal stress response system (Type BV+ST models)—with agents endowed with the latter model significantly outperforming the Type BV model and Control across all viability-related metrics.

Taken together, we found support for our hypothesis (H1) that “social buffering” can improve the viability of a small group of artificial agents with affective social bonds, through the social regulation of adaptive “stress” mechanisms. However, in contrast to our prediction that we would see larger wellbeing improvements associated with oxytocin’s modulation of social salience, we found that the magnitude in wellbeing improvements was significantly greater between the two groups endowed with the different “social buffering” mechanisms (Type BV vs. Type BV+ST models) than between the groups with no “social buffering” mechanisms and those endowed with a single “social buffering” mechanism (Control vs. Type BV models).

Furthermore, we found that the efficacy of these “social buffering” mechanisms was not consistent between environmental and social conditions; suggesting that the benefits associated with “social buffering” may not be generalisable. Rather, the (long-term) affective perception and interactions with social support, and therefore its effects on regulating stress and improving viability, was significantly affected by the amount of positive, early-stage bond partner interactions, as well as the degree of the physical and social challenges for bonded agents. We found how early-stage bond partner interaction—and thus the long-term affective perception of the social and physical environment—was also significantly affected by the types of stress-regulatory mechanisms that bonded agents were endowed with.

Despite being detrimental to agent performance in some scenarios, we found how a stress-induced hormone (CT) can provide adaptive viability advantages (with respect to regulating a homeostatically-controlled internal physiology) for artificial agents, permitting agents to dynamically adapt physiology and behaviour across numerous contexts. The degree to which “stress” was adaptive (as opposed to maladaptive) was contextually-dependent on both the challenge of the physical environment as well as the (perceived) availability of affective social support. We argue that researchers of embodied, socially-adaptive agent models should consider such affective, stress-related mechanisms as a low-level adaptive mechanism.

The contextual differences in our findings suggest that the (efficacy of the) stress-reducing effects of social support may not be a universal phenomenon among social (natural and artificial) agents, but rather dependent on the wider social and physical environment, as well as an agent’s own development of affective systems. For natural agents, our results can guide future studies in better understand some of the contexts that underpin the inconsistent stress-reducing effects of social support (Abbott et al., 2003). For artificial (social) agents, these findings have implications on the future development of their socially-adaptive models. Rather than a general model of (social) adaptation, future approaches may need to consider the specific environmental or social environments that these artificial agents will be required to adapt to. Below, we focus on several key findings below, supplementing the quantitative results presented above with qualitative analysis.

### 4.1 Early-Stage Social Interactions Affects Long-Term Affective Perception of Social Support and Long-Term Survival

We found how both the long-term affective perception of social support (in non-control groups) and survival time (Life Length) was strongly correlated with early-stage positive social interaction (*Grooming*) between bond partners (Table 7); underpinning the long-term efficacy of the “social buffering” effects of (affective) social support (via stress reduction, Figure 9). These results highlight a significant role of adequate, early-stage positive social interactions in the long-term development of affective (social) perception of social bond partners: potentially affecting the efficacy of their stress-reducing (“social buffering”) effects. Specifically, that there was a strong relationship between the number of early-stage positive social interactions between bonded agents, an agent’s affective perception of bond partners, and their survival time (i.e. their ability to maintain homeostatic stability).

These dynamics support discussions from both neuroscience (Gunnar and Hostinar, 2015) and psychology (Landry et al., 2001) regarding the implications of early-stage positive social interaction on the long-term social and affective development in social agents (including humans): with oxytocin a likely mediator in this affective development (Carter, 2014). Similarly, the lack of (early-stage) social interactions has been found to have adverse effects on long-term affective development—and even the onset of social and affective disorders—across numerous animal models (see (Mumtaz et al., 2018) for a review). The results from our embodied model further strengthens these suggestions.

However, this association was only found in the models where oxytocin also modulated internal stress tolerances (Type BV+ST), and not in groups where oxytocin only improved bond partner valence. These results may therefore give insight into some of oxytocin’s mechanisms in biology which contribute to the affective development of social agents, and we predict that it is this “buffering” of the internal stress tolerance system (i.e. such as the autonomic nervous system) that may play a significant role in the socially-affective development in biological agents Future work using biological models should look at empirically testing this hypothesis.

### 4.2 Oxytocin-Mediated “Social Buffering” Facilitates an Affective “Anticipation” of Stressful Conditions

**FIGURE 12:**
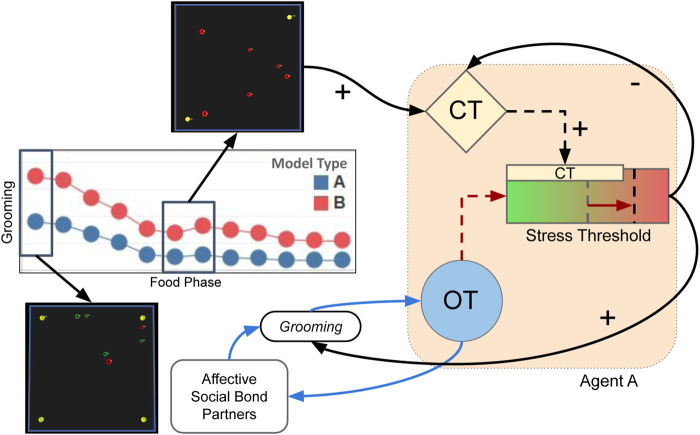
Illustration for how early-stage socio-positive interaction in Type BV+ST, but not Type BV, models shaped long-term affective perception and the long-term efficacy of “social buffering.” Grooming in early stages (when food was more abundant) releases OT in Agent A, which reduces the perception of external stress (Eq. 8) and therefore reducing CT release. In Type BV+ST models, OT adapts the internal Stress Tolerance, permitting higher amounts of CT to be tolerated before undergoing “stress”. In later food phases (in SEA and EXT conditions), when food becomes scarcer and external stress leads to increased CT release, CT levels were less likely to transgress the “buffered” Stress Tolerance value. This resulted in reductions in socio-negative interactions, and increased socio-positive interactions, between bond partners: which underpinned further OT release, improving valence of social support, and improved the long-term efficacy of “social buffering” effects. + denotes increase of hormone or behaviour. - denotes a decrease.

A second observation of agents endowed with the Type BV+ST model (where OT “buffered” their stress tolerance) was that this mechanism underpinned a positive affective feedback loop between physiological adaptation and socio-positive interactions (Figure 12). We attempt to explain the causal interactions underpinning this affective feedback loop, with references to our quantitative results where appropriate.

Early-stage *Grooming* between bond partners (i.e., when food was more abundant, Figure 5) as described in Section 4.1 permitted OT to stay elevated for bonded agents (Figure 8). Elevated OT, which adapted the *Stress Tolerance* (i.e., the activation level of the “sympathetic nervous system,” Eq. 15; Figure 10) of these bonded agents, then permitted those agents to tolerate more stress (CT) before becoming “stressed”. In dynamic environments (SEA and EXT), when the physical environment changed (i.e., when food availability decreases at various rates over time), the increased environmental stress increases CT levels (Figure 9). However, as OT levels modulated internal stress tolerances (*θ*_ST_) *before* these environmental changes occurred, agents did not become “stressed.” Instead, they continued to perform socio-positive interactions (as opposed to socio-negative interactions) between each other. In other words, agents were able to maintain strong affective relationships and elevated OT levels during periods of “good” environmental conditions, which “buffered” their Stress Tolerance before environments changed: as a type of “anticipatory” process to withstand the upcoming environmental changes.

Conversely, in Type BV models where OT did not adapt this internal Stress Tolerance, bonded agents reported lower OT levels (Type BV: 0.37–0.51, Type BV+ST: 0.40–0.74), increased CT levels (0.28–0.66 vs. 0.22–0.60), and higher absolute intra-bond *Aggression* numbers (810 vs. 296). These physiological and behavioural consequences of not adapting the stress tolerance limited the long-term efficacy of social support on reducing stress.

For Type BV+ST agents, the stress-reducing effects of OT-mediated social support resulted in increased OT levels for socially-supported agents, which promoted further adaptation of the stress response and prosocial behaviours which further promoted OT release and maintained strong affective relationships with bond partners.

We suggest that these allostatic-type mechanisms, mediated by oxytocin, constitute a type of low-level, affect-based anticipatory adaptation of an agent’s internal model prior to the onset of future (unseen) stressful or threatening conditions, improving viability at the agent and group level.

This affective feedback loop mirrors similar suggestions from biology and neuroscience: that OT’s effects on stress-regulation may be facilitated (in part) by its positive feedback loop on seeking prosocial interactions (Taylor, 2006; Insel and Young, 2001). However, in biological systems, reward-based dopaminergic mechanisms have previously been suggested to play a role in these effects (Bielsky and Young, 2004; Nelson and Panksepp, 1998). Our results found these effects to emerge in absence of such systems. Considering our biologically-grounded approach to modelling these hormonal mechanisms and behaviours (Section 2) and the findings of this study, we predict that in natural systems (including humans), OT mechanisms may play a more direct role on affiliative behaviours, independent of reward-based mechanisms.

### 4.3 Early-Stage Social Interaction, Affective Development, and “Tend-and-Befriend” Behaviours for Long-Term Stress Regulation

Following observations in ethology which finds associations with food scarcity and reduced intra-group grooming interactions (i.e. more competition (Yamakoshi, 2004; Lehmann et al., 2007), we had expected to find reduced intra-bond *Grooming* during periods of “poorer” food availability (i.e. as environmental stress increased: Table 4). However, this was only found for bonded agents endowed with the Type BV model (Figure 11). In contrast, Type BV+ST groups reported increases in socio-positive interactions (*Grooming*) and reduced *Aggression*, even during periods where the external environment increased in challenged.

As we found in our previous study (Khan et al., 2020), the different social behaviours exhibited during stressful conditions bear similarities to (the behavioural components of) the “fight-or-flight” and “tend-and-befriend” theories. Briefly, “fight-or-flight” (Cannon, 1929) proposes that, in response to a threatening or stressful situation, social agents can either become aggressive towards others or withdraw from the situation. More recently, the “tend-and-befriend” hypothesis (Taylor, 2006) suggests that social agents (including humans) seek positive, affiliative interactions during stressful conditions in order to facilitate long-term survival.

We propose that the “fight-or-flight” behaviours seen in Type BV agents was a stress-reducing behaviour performed in the absence of adequate anticipation of stressful conditions—a “reactive” behaviour in absence of any “buffering” of stress tolerance—while the “tend-and-befriend” behaviours seen from agents endowed with the Type BV+ST models were associated with agents that also engaged in early-stage positive social interactions (Section 4.1). As we suggested above, these early-stage social interactions were associated with elevated OT levels and the long-term affective regulation of stress mechanisms, which we suggest underpinned a type of socially-mediated “affective development” in these agents.

Given oxytocin’s implications on nurturing, “tend-and-befriend” behaviours across species (Taylor, 2006; Carter, 2014; Ross and Young, 2009), our findings may lend further support to the hypothesis that these (long-term) stress-coping behaviours develop as a result of positive social interactions in early life stages; which may underpin the appropriate development of an “affective” system via OT mechanisms. In addition, the “tend-and-befriend” hypothesis has also been considered a more “maternal” behaviour in natural systems (Taylor, 2006), where both elevated levels of OT and female reproductive hormones have been implicated in mediating these social behaviours. This suggestion is supported by our own results, with the increased OT levels we had observed in our Type BV+ST models (Figure 8) correlating with early-stage (and overall) socio-positive interactions (Table 6).

Taken together, these findings suggest that oxytocin may play a significant role in early-stage affective development, and we predict that its effects on developing (or adapting) an individual’s tolerance to (physiological) stress (via its effects on the autonomic nervous system) may be a key contributor to the different stress-regulating behaviours (“fight-or-flight” vs. “tend-and-befriend”) exhibited during stressful situations. Future investigations should consider focusing on the effects of oxytocin, in absence of other hormones (such as testosterone and oestrogen which have been implicated in these behaviours) in biological systems.

While these mechanisms may be difficult to study in biological systems, we propose that embodied computational models (such as the model presented in this paper) can be used to appropriately abstract and test these hypothesised biological mechanisms *in silico*. For adaptive models interested in human–robot (or robot–robot) interaction, these findings also highlight how the temporal nature of positive (social) behaviours can play a significant role in the long-term efficacy of behavioural adaptation.

### 4.4 Hormones as Embodied, Allostatic Biomarkers of Contextual Information for (Artificial) Agents

We argue that the hormonal mechanisms that we have modelled can be considered embodied physiological “biomarkers” by being able to dynamically encode (historical and current) environmental information. Further, we suggest that these minimal, low-level mechanisms can complement (and even challenge) existing (biologically-inspired) approaches to embodied adaptive systems, as a computationally-efficient approach to integrating numerous points of (contextual) information. The encoding of this information via (simple) hormonal mechanisms can be considered a type of functional affective state—aggregating multiple, diverse sources of information down to a small subset of embodied signals—that can then play an adaptive or predictive role in embodied, socially-adaptive agent models. Here, we present our view on how both of the hormones in our agent model appropriately encoded contextual information about internal and external (both physical and social) environments.

Cortisol’s release function (Equation 8) accounts for the (perceived) internal physiological error signals—which can be considered a low-level interoceptive signal of physiological states [Schulz and Vögele, 2015]—and the “uncertainty” (specifically through the perceived absence of resources), of the external environment (Sapolsky, 2004). Simply put, cortisol can be considered an embodied physiological signal that aggregates the current information about the (perceived) internal and external environments, with a parameter (**w**) that determines its sensitivity to perceived changes.

Our modelling of oxytocin—released as a function of positive social interaction—can be considered an internal signal that represents the (perceived) level of (historical) positive social support in a small society. Here, rather than modelling discrete, computational models of memory as previous approaches have done (Leite et al., 2014; Ho et al., 2009) and given its modulatory role on the formation (and recall) of social memory and social learning in biological systems (Neumann, 2008), we present our direct approach towards modelling “social memory” as a low-level, biologically-inspired approach that can capture the dynamics of this higher-order (cognitive) function in our embodied agent model.

In addition, since CT had a direct effect on adapting a homeostatically-controlled need (*Energy*, Table 1), and OT (directly and indirectly) acted on systems that contribute to reactive or anticipatory social behaviours (Section 4.3), these basic hormones sufficiently capture “allostatic” mechanisms (Schulkin, 2011; Sterling, 2020). For OT specifically, this mirrors more recent suggestions from cognitive science (Quintana and Guastella, 2020), suggesting that the biological hormone (oxytocin) should be considered a hormone that underpins allostatic adaptation, rather than only having a regulatory role (or roles) on social behaviours (Bethlehem et al., 2014).

This minimal approach stands in direct contrast to similar approaches towards embodied (socially-)adaptive models. Most notable are the more recent approaches that are built on the predictive processing (or active inference) framework (for example, (Ahmadi and Tani, 2019; Murata et al., 2015; Schillaci et al., 2020; Park et al., 2017)) that often focus on the modelling of higher-order cognitive function. Such approaches often take computationally-expensive approaches (i.e. built on deep neural networks), and either do not update their models to account for contextual interactions during runtime (i.e. all training is done offline) or perform these updates (retraining the model) as a batch process. While such models are naturally designed to exploit the computational resources available (and thus perform well in their respective experimental paradigms) we argue that this puts a (computational) limit on the generalisability of these approaches, for both artificial and biological systems.

We propose that our computationally-cheaper abstraction of simple hormonal mechanisms is sufficient in capturing historical (prior) and current, contextual information relevant to the (perceived) internal, external and social environments, and it is this information that permits physiological and behavioural adaptation (of our homeostatically-controlled agent model). We have demonstrated that this bottom-up, (biologically-inspired) approach can adequately capture features of “affective cognition” (Cañamero, 2019) and can play a significant role in socially-adaptive embodied agent models: overcoming some of the limitations of previous approaches and proposing a more generalisable approach for future models. We therefore suggest that approaches towards embodied, (socially-)adaptive models—and in particular biologically-inspired approaches, or those working with computational or temporal constraints (for example, physical agents interacting with the world in real-time; with limited onboard computational resources)—should consider accounting for similar low-level, hormonal mechanisms as part of their adaptive models to capture some dynamics of these higher-order cognitive mechanisms.

## 5 Concluding Remarks

In this paper, we have investigated how some of the hypothesised, hormonal (oxytocin) mechanisms associated with the “social buffering” phenomenon affects the wellbeing and social behaviours of agents in a small society. Building on previous findings, we had hypothesised that two of oxytocin’s stress-regulating effects (improving the valence of social support, and adapting an internal tolerance to physiological stress) would provide significant advantages to long-term wellbeing of agents across numerous dynamic physical and social environments.

We used a simulated agent model to investigate our hypotheses in a small, rank-based society of six artificial agents, whose goal was to “survive” by maintaining the stability of their internal environment through physical and social behaviours, and investigated this across a number of dynamic environments. We modelled some of the effects related to “stress” and the “social buffering” phenomenon through simulated hormones (cortisol and oxytocin) and incrementally accounted for their effects in our agent model.

Our results found how these hormonally-mediated, stress-regulating effects the “social buffering” phenomonon provided significant advantages to the wellbeing of agents with affective social support. However, the long-term efficacy of these effects was dependent on the contextual perception of affective social support (affected by the amount of early-stage social interaction), the environmental contexts, and the precise hormonal mechanism(s) accounted for in the agent model. Based on the improvements observed from the combination of multiple hormonal effects in the agent model, our results suggest that for artificial agents, and potentially in biological agents, the effects of social support on regulating stress may be multi-faceted in nature.

For natural (social) agents, our experiments show support for the suggestions that early-stage positive social interactions can play a significant role in long-term affective and social development through oxytocin-related mechanisms, which has implications on long-term behavioural choices for social agents. For (socially-)affective artificial agents, we argue that viability across dynamic social and physical environments can be improved via numerous mechanisms related to “social buffering”. We also argue that (affective) “stress” can play a significant role as an adaptive mechanism to promote long-term wellbeing for (artificial) agents through the adaptation of physiology and behaviours. Subsequently, we suggest that socially-situated embodied agent models consider these respective mechanisms as part of their approaches. Finally, we argue that low-level, hormonal mechanisms can be used as embodied “biomarkers” to minimally-encode interoceptive and exteroceptive signals which can be used as mechanisms for real-time, affect-based contextual decision-making—addressing potential computational limitations of similar approaches—and we suggest that future embodied (socially-adaptive) models should consider these low-level mechanisms as part of their approaches to long-term social adaptation.

## 6 Limitations and Future Work

To the best of our knowledge, our work is currently unique in studying “social buffering” (and mechanisms of social allostasis) using embodied agent models, and our findings and subsequent discussion may be limited by this lack of related work in the field. While our model has used empirically-determined parameter tuning in building the model and testing our hypotheses in our simulation environment, we recognise that such approaches may suffer from overfitting the experimental paradigm. To verify our model, we would like to see such systems replicated and extended to additional artificial agent societies or physically-embodied agents: either in human-robot interactions, or in small, multi-agent systems.

In comparison to both biological agents and approaches to (socially-)adaptive behaviour in HRI, our present agent model—limited to only two internal “physiological” needs and two (types of) behaviours—might appear too simple. This comment also applies to the simulation environment (i.e. the artificial ecology) that the experiments were conducted in. As we discuss in Section 2, this minimally-complex model was a purposeful decision: to maintain an appropriate balance between a model that allows for complex, adaptive behaviours, while remaining simple enough to analyse. Depending on the research questions investigated in the future, and following our incremental design and methodology, further work would consider extending this agent model—for instance, by adding an additional physiological variable that needs to be regulated, or an additional agent behaviour—or to investigate different environment dynamics (i.e. changing the timings of food “seasonality”, or making such environmental processes more stochastic) to further understand the contextual nature of “social buffering” as it may relate to biological systems. This future work may also consider investigating additional hypothesised systems—such as reward-based mechanisms associated in positive social interactions—to better understand their role in (the efficacy of) “social buffering” and improving the long-term adaptability of agents in artificial (and natural) systems.

Related to this point is that, biological societies significantly vary in size and connectedness (see, for instance, primate societies (Dunbar et al., 2018). It is currently unknown if, and how, our current findings (using a society of six agents) may scale up to larger social groups, different groups of “bonded” individuals in a sub-group, or the effects of dynamic social structures in higher-order biological systems (such as fission-fusion dynamics). As part of our future work, we intend on investigating these models in both larger social groups, and dynamic sizes of “bonded” agents, in an effort to replicate (human and non-human) primate social structures. However, increases in social group size comes at computational costs: 1) the statistical non-independance of individual agent data requires more observations for statistical analysis (i.e. the “scaling” problem (Kenny et al., 2002)) and 2) the increased complexity in capturing the non-linear interactions in such systems (the “dynamics” problem (Hoey et al., 2018)). Such scaling up of group sizes, therefore, should be done so with caution.

One ongoing question with this type of computational modelling, particularly in embodied systems, is, “how generalisable is (this model) to other (artificial and biological) systems?“. We argue that our modelling of these hormonal dynamics, grounded in ethological and neuroscience literature (Table 2) have maintained an appropriate level of abstraction which can promote the generalisability of our results. To validate the work undertaken, our future work aims to investigate any potential relationships between the hormonal dynamics and agent behaviours from our own results with those from the neuroscience and ethology literature.

## Data Availability

The datasets and simulation models presented in this study can be found online at http://data.imytk.co.uk.
