# Supplementary material for: The Long-Term Efficacy of “Social Buffering” in Artificial Social Agents: Contextual Affective Perception Matters
Source: Front Robot AI. 2022 Sep 15;9:699573. doi: 10.3389/frobt.2022.699573 (PMC9520257; doi:10.3389/frobt.2022.699573)
Supplement: Supplementary file 1 [file DataSheet1.ZIP › behaviour_videos_readme.rtf]

behaviours_videos.mov is a quick recording of the simulation environment, and intended to give some visual examples of the Touch and Eating behaviours that our agents perform.Agents are represented as the green or red discs which are moving through the environment. Food resources are the static yellow spheres in each of the four corners of the world.Agents have two behaviours - Touch other agents and Eat food. Both behaviours can be seen in this video.Touch can be seen when two agents are in close proximity to each other and both agents flash white. Examples are at 13s (A1+A3), 14s (A2+A5), 20s (A1+A2), 30s (A3+A6, and A1+A6), etc. Eating food can be seen when agents approach and stop at a food resource - the food resource turns orange (for visualisation purposes), and the agents stop to take “bites” of the food. Examples are seen at 7s (A5), 11s (A1 and A3), 26s (A2). Longer eating cycles are seen at 53s (A2), 1:02 (A4), 1:08 (A5). The number above the food resource represents the amount of “nutrition” remaining in that resource. When agents have completed their eating behaviour, they move away from the resource. Multiple agents can eat at the same resource (for example, at 1:28, A1+A2).
