# Supplementary figures and images for: The Long-Term Efficacy of “Social Buffering” in Artificial Social Agents: Contextual Affective Perception Matters
Source: Front Robot AI. 2022 Sep 15;9:699573. doi: 10.3389/frobt.2022.699573 (PMC9520257; doi:10.3389/frobt.2022.699573)

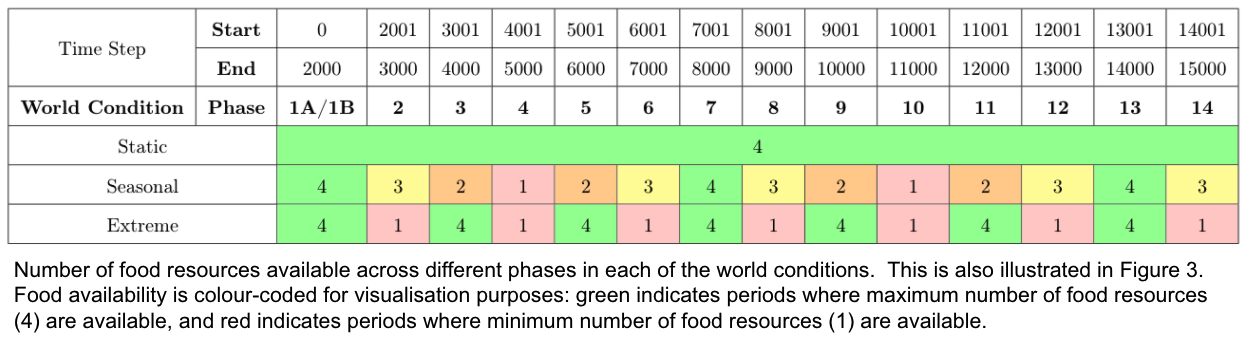

Supplement: Supplementary file 2 [file Image1.PNG]
